# Western medical acupuncture in a group setting for knee osteoarthritis: results of a pilot randomised controlled trial

**DOI:** 10.1186/s40814-016-0051-5

**Published:** 2016-02-16

**Authors:** Adrian White, Liz Tough, Vicky Eyre, Jane Vickery, Anthea Asprey, Cath Quinn, Fiona Warren, Colin Pritchard, Nadine E. Foster, Rod S. Taylor, Martin Underwood, Paul Dieppe

**Affiliations:** 1 Primary Care, Plymouth University Peninsula Schools of Medicine and Dentistry, Room 14, ITTC Building, Plymouth Science Park, Plymouth, PL6 8BX UK; 2 Peninsula CTU, Plymouth University Peninsula Schools of Medicine and Dentistry, Plymouth, UK; 3 Primary Care, University of Exeter Medical School, Exeter, UK; 4 Royal Cornwall Hospitals NHS trust, Truro, UK; 5 Arthritis Research UK Primary Care Centre, Research Institute of Primary Care and Health Sciences, Keele University, Keele, UK; 6 Warwick Medical School, Coventry, UK

**Keywords:** Primary health care, Knee osteoarthritis, Randomised controlled trial, Acupuncture, Healthcare delivery, Pilot project

## Abstract

**Background:**

Evidence suggests acupuncture may be effective for treating the symptoms of knee osteoarthritis. Offering this in a group setting may offer cost savings. The aim of this study was to establish the feasibility of a definitive trial to assess the clinical and cost-effectiveness of Western medical acupuncture given in groups, or given individually, for adults with severe knee pain attributable to osteoarthritis.

**Methods:**

A pilot randomised controlled trial (RCT) was conducted. Participants were recruited from seven general practices in Plymouth, Devon. Acupuncture was provided, at a dosage that increased up to and including electroacupuncture if no pain relief was reported, by one experienced acupuncturist in a community clinic. Potentially eligible adults aged at least 45 years with knee osteoarthritis were identified from practice registers, screened and randomised to either: (1) standardised advice and exercise booklet alone (‘standard’); (2) booklet plus group acupuncture (‘group’); and (3) booklet plus individual acupuncture (‘individual’). Both acupuncture arms received up to ten treatments over 12 weeks.

Recruitment, retention and data completion rates were recorded, and participants completed questionnaires on acceptability. We collected pain, stiffness and function data (using the Western Ontario McMaster Universities Osteoarthritis Index; WOMAC) and general health (EQ-5D) and economic measures at baseline and 14 weeks post-randomisation.

**Results:**

We screened 149 people and randomised 60 (40 %), 20 per arm. The overall 14 week follow-up rate was 77 %, but only 70 % in the ‘standard’ group; 4.1 % of data points were missing. The study was acceptable to participants.

Changes in WOMAC pain score (intention to treat complete case analysis) from baseline to 14 week follow-up were: ‘standard’, 0.4 (95 % confidence interval (CI) −1.4, 2.2, *n* = 14); ‘group’ −3.2 (95 % CI −5.1, −1.4, *n* = 17); ‘individual’ −2.4 (95 % CI −4.1, −0.7, *n* = 15).

**Conclusions:**

A definitive three-arm trial is feasible. Further follow-up reminders, minimum data collection and incentives should be considered to improve participant retention in the follow-up processes in the standardised advice and exercise booklet arm.

**Trial registration:**

ISRCTN05305406

**Electronic supplementary material:**

The online version of this article (doi:10.1186/s40814-016-0051-5) contains supplementary material, which is available to authorized users.

## Background

Severe knee pain, mostly caused by osteoarthritis (OA), affects about 17 % of the UK population aged over 50 years and is the principal cause of disability in the elderly [1–3]. Knee OA reduces quality of life and may lead to depression and social isolation [4, 5]. Management of OA is currently suboptimal [6], and half of patients have inadequate pain relief [7]. Not only do comorbidities often limit options but also interventions such as exercise have modest effects [8] that decline over time [9]. Joint replacement is costly, may not be effective [10], and is often inappropriate or unacceptable to patients [11]. Thus, a ‘treatment gap’ is identified [12], and simply waiting until the joint is ready for replacement is not optimal care [13].

Knee pain accounts for over 250,000 acupuncture consultations each year in the UK [14], half of these in the National Health Service (NHS) where it is delivered by trained general practitioners (GPs), physiotherapists, nurses and acupuncture practitioners. A course of acupuncture can produce sustained changes in the nervous system (neuromodulation) [15]. Six high quality randomised controlled trials (RCTs) show that acupuncture is an effective treatment for OA pain [16] and function [17]. Indeed, meta-analysis shows it as one of the most effective of conservative treatments [18]. It has statistically significant effects beyond placebo [16] and is recommended for treatment of knee OA by several guidelines [19] and [20].

Acupuncture has been shown to be a cost-effective adjunct to physiotherapy-led advice and exercises for knee pain [21]. To reduce acquisition costs, a nurse-led group treatment setting was introduced in one UK general practice for patients considering surgical referral for OA knee pain [22]. The group setting may bring extra benefits from normalising symptoms, sharing information, and encouraging attendance [23]. Patients report pain relief, satisfaction, and deferred referral for surgery [22]. Group acupuncture clearly justifies further investigation.

We plan a definitive randomised trial to test whether group acupuncture, added to standardised care consisting of advice to exercise, is an effective treatment for patients with severe knee OA in general practice. We here report a pilot randomised trial with the key objectives of determining rates of recruitment, retention, treatment attendance, and data completion; acceptability of the trial’s components; and to inform sample size estimation.

## Methods

A pilot randomised controlled trial (RCT) was conducted with three parallel arms: standardised advice and exercise booklet, the booklet plus group acupuncture, and the booklet plus individual acupuncture. This allowed us to explore practicalities of a study comparing the two acupuncture interventions separately with standardised advice booklet and with each other. The design follows NICE (National Institute for Health and Care Excellence) recommendations on OA by including the very elderly, testing therapy combinations, and identifying subsets of patients likely to respond [24].

The study setting was primary care in Plymouth, Devon. Ethics approval for the study was granted by NRES Committee South West—Cornwall and Plymouth, ref 11/SW/0277.

A sample size of 60 was chosen as adequate for a pilot RCT [25]. Seven participating GP research practices searched their databases for patients aged at least 45 years with Read codes for ‘osteoarthritis’, ‘knee’ (though rarely coded), and ‘not dementia’. GPs screened out patients considered unsuitable, and the remainder was sent a letter introducing the study. Those interested replied directly to the research centre, were screened for inclusion and exclusion criteria (see box) by telephone and, if potentially eligible, sent information leaflets (see Additional file 1) and a screening questionnaire (Oxford Knee Score, OKS). Those still interested and eligible were invited to the research centre to give informed consent and complete baseline questionnaires and physical examination by the research nurse.

### Inclusion and exclusion criteria

Participants had to be aged 45 years or over, to meet the American College of Rheumatology (ACR) clinical criteria for osteoarthritis [5], and to have an OKS (Oxford Knee Score) of ≤28/48 at screening (low scores worst: 28 represents one SD above the mean OKS in patients undergoing knee replacement surgery [6]). Participants were excluded if they had a history of undiagnosed or severe bleeding disorder (contraindication of acupuncture), steroid injection or acupuncture to either knee in the last 2 months, and hyaluronic acid injection, arthroscopy or serious injury to either knee in last 6 months; were currently referred for, or had, replacement surgery in the index knee; had a clinical diagnosis of severe OA of the ipsilateral hip; had concurrent medical conditions which would impair participation; or were currently participating in any other interventional clinical trial.

### Randomisation and blinding

Immediately following baseline assessment, participants were allocated to treatment arm by means of a secure, web-based programme accessed by the research nurse. Allocation was minimised on two factors: body mass index (BMI) (BMI ≤30, BMI >30) and OKS (OKS ≤20, OKS >20), and to ensure allocation concealment, the minimisation algorithm maintained a stochastic element. The web-based allocation was developed and supported by the UK Clinical Research Collaboration (UKCRC) registered Peninsula Clinical Trials Unit (PenCTU).

The statistician conducted the data analysis blinded to allocation. Given the nature of acupuncture interventions, it was not possible to blind the participants, other research staff, or physiotherapist delivering the acupuncture.

### Interventions

Participants in all three arms received a standardised advice and exercise booklet. The standardised care arm (‘standard’) had the booklet alone and thus served as the control arm, one arm also had group acupuncture (‘group’), and the third arm also had individual acupuncture (‘individual’). The group acupuncture intervention was based on the nurse-led primary care model [26], and provided at no cost to patients.

Standardised care: the advice and exercise booklet (see Additional file 2), containing information about OA and pain management, and advice on standardised exercises and weight loss if appropriate, was purpose-designed, based on publicly available booklets (e.g. from Arthritis Research UK) but avoiding any mention of acupuncture. The research nurse gave the booklet to all participants immediately after treatment allocation, encouraged them to read it and engage in the exercises, and advised them to telephone the trial coordinator if they had questions. No instruction or advice on the use of other exercise(s) was given to participants.

Acupuncture: Participants allocated to acupuncture were also given an information leaflet about it (see Additional file 3) and subsequently telephoned by the trial acupuncturist to arrange the first appointment. A single acupuncturist provided group and individual acupuncture in a Primary Care Trust healthcare centre. Apart from the setting (group/individual), we aimed to deliver treatment to both arms in as similar manner as possible. Group size was minimum two and maximum six. New participants joined the next available group session and could see different participants during their course of treatment.

The acupuncture protocol (see Additional file 4) following a westernised approach [27] was based on one used by a GP clinic [26] and approved by tutors of the British Medical Acupuncture Society. Up to eight common points (four local standard points and up to four additional tender points) were used. The acupuncture dose was titrated upwards at each appointment if the participant did not report any improvement in symptoms: strength of stimulation escalated to include electroacupuncture (electroacupuncture (EA) at alternating 2/80 Hz causing strong sensation or muscle contraction) and increased duration up to 30 min. Both knees were treated if painful, though the worse knee nominated by the participant was evaluated. The acupuncture protocol permitted six to ten sessions in 12 weeks, initially weekly. Treatment was stopped at week 6 if a pain rating scale showed no reduction from baseline. No additional interventions by the acupuncturist were allowed.

The study acupuncturist was a registered physiotherapist with eight years’ acupuncture experience. He was instructed not to advise participants about exercise or the booklet.

### Baseline data, outcomes, and measures

Feasibility objectives were addressed by recording numbers of participants at each stage of the recruitment process, numbers of acupuncture sessions attended, and, at 14 weeks, participant-reported use of exercises and numbers of participants providing data and percentage of missing data.

Socioeconomic and health data, including knee pain location [28] and current use of treatments including exercise (as defined by the participant) and aids, were collected by the nurse at baseline. The primary clinical outcome planned for the main trial is the pain subscore of WOMAC (Western Ontario & McMaster Index®) [29]. Data were collected at baseline attendance and at 14 weeks by mail, with one postal reminder.

We used the OKS questionnaire on pain and function as the screening questionnaire and secondary outcome [30]. Our planned health-utility outcome for economic analysis is the EuroQol five dimension quality of life instrument (EQ-5D). We piloted a purpose-designed questionnaire to assess use of social and health resources, and we captured costs of acupuncture from invoices. We did not include the cost of the standardised advice and exercise booklet since it was given free to all participants.

Other variables were assessed by purpose-designed questionnaires (see Additional file 5). At baseline, we assessed physical activity, analgesic use, global troublesomeness of the knee problem, and expectations of exercise and acupuncture. At 14 weeks, we assessed global perceived change, adverse events, and reported use of booklet exercises; adverse events of acupuncture were collected at each treatment visit. Completion of study outcomes and procedures was regularly appraised.

All participants were invited to complete commentaries at baseline, 6 and 14 weeks, and, for acupuncture groups, after the second consultation. These contained 18 questions on acceptability (see Additional file 6), with responses on five-point Likert-type scales (very dissatisfied to very satisfied). Responses were also used to select interviewees in a nested qualitative study exploring reasons for joining or leaving the study, to be reported separately.

To pilot methods of identifying treatment responders, we tested pressure pain threshold and brush-stroke allodynia (to be reported elsewhere). Procedures and outcome measures were evaluated for optimal performance and revised appropriately.

### Analysis

**Fig. 1:**
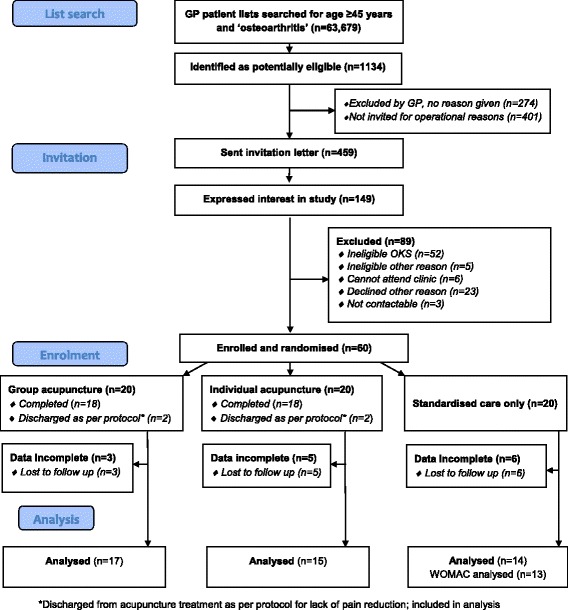
Study flow diagram

Recruitment was assessed by constructing a consolidated standards of reporting trial (CONSORT)-type flow diagram (Fig. 1), retention from 14 week responses, treatment attendance from clinic records, and data completion by percent valid response to individual items. Given the feasibility objectives of this pilot trial, outcome results were reported descriptively. The mean and standard deviation (SD) were reported at baseline and 14 weeks’ follow-up by arm, as well as the mean change in WOMAC pain score (14 weeks minus baseline), with its associated 95 % confidence interval, for each arm. No between group inferential comparisons were planned. All analyses were based on the intention-to-treat principle (i.e. according to randomisation) using complete case data only and were performed using Stata v12.

## Results

### Recruitment, retention, treatment adherence, and data completion

Recruitment of seven practices and 60 participants was achieved on schedule between January and October 2012. From 63,679 people registered at participating practices, 1134 were identified as potentially eligible (mean per practice 1.8 %, range 0.3 to 3.4 %) see Fig. 1. Interest was better than anticipated; therefore, only 459 (53 % of available) patients were invited, of whom 13 % were enrolled.

All 40 participants allocated to acupuncture attended at least four sessions. Four participants (two in each arm) were discharged after six sessions for lack of response. Mean (SD) sessions attended were ‘group’ 8.5 (1.4) and ‘individual’ 8.4 (1.5). In acupuncture groups, each participant usually met about four others during each treatment session. Electroacupuncture was used for most participants: 16 participants in ‘group’ and 18 in ‘individual’. At 14 weeks, the numbers reporting use of exercises at least 3 days a week were ‘group’ 10/17 (59 %), ‘individual’ 10/15 (67 %), and ‘standard’ 8/14 (57 %).

A total of 14 (23 %) participants did not provide any follow-up data, one of whom dropped out for planned surgery; no meaningful differences in baseline characteristics between participants lost to follow-up, and the overall sample were identified. Two further WOMAC questionnaires were not fully analysable. Loss to follow up at 14 weeks was highest (6/20, 30 %) in the ‘standard’ arm.

The amount of outcome data missing from returned questionnaires at follow-up was small, the highest being for WOMAC where 4.1 % of items were not validly completed.

### Acceptability of trial components

Responses are shown in Additional file 6. For selected questions, the proportions of responses in the two most favourable options were: good experience overall, 36/50 (72 %); pleased to have volunteered, 42/45 (93 %); had not considered dropping out, 38/44 (86 %); and willing to recommend the study to a friend, 42/44 (95 %). Acceptability of the group acupuncture clinic was 17/19 (89 %) compared with 20/20 (100 %) for the individual clinic.

### Revisions to procedures and outcome measures

We initially included the OKS and screening questionnaire with the study invitation, generating 117/413 (28 %) responses (range between practices 20–44 %). Attempting to improve recruitment, we sent a letter alone in the last two practices, generating 25/46 (54 %, range 53–67 %) responses. One question on the use of social and health resources was revised to improve the response.

We noted three problems: 13 participants met the inclusion criterion of OKS ≤28 at screening but scored >28 at baseline attendance. At baseline, participants had difficulty in scoring their expectation of the effect of exercise and of acupuncture and answering the questionnaire on physical activity (see comments in Additional file 5). The scoring system for knee pain location method [28] was too complex to be workable, and we recommend additional staff training in future studies.

### Study population

Participants’ mean age was 65 years (SD 9.4, range 45–91), and mean BMI was 31.8 (SD 6.2, range 23.4–59.8). The three trial arms were well balanced by age and BMI, with a greater proportion of males in the ‘individual’ arm (Table 1). Few participants were getting much relief from non-steroidal anti-inflammatory drugs (NSAIDs) or exercise, though 35 (58 %) reported not having been advised (e.g. by GP, nurse or physiotherapist) to do exercises for their pain. Forty-nine (82 %) participants had bilateral pain, and 40 (67 %) had their pain for over 5 years. One used a wheelchair, and 24 used a walking-stick. Mean (SD) WOMAC scores were pain 9.9 (SD 2.9) and total WOMAC 49.4 (12.8).

**Table 1:** Baseline characteristics of the three arms

	‘group’ (*N* = 20)	‘individual’ (*N* = 20)	‘standard’ (*N* = 20)
Male, *n* (%)	10 (50)	12 (60)	10 (50)
Age in years (mean, SD)	64.7 (7.7)	65.1 (9.9)	64.9 (10.8)
BMI (mean, SD)	32.0 (8.2)	31.7 (5.1)	31.7 (5.3)
Main activity, *n* (%)			
Employed	7 (35)	7 (35)	9 (45)
Housework	0 (0)	0 (0)	1 (5)
Retired	13 (65)	12 (60)	9 (45)
Other	0 (0)	1 (0.5)	1 (5)
Pain elsewhere, *n* (%)	12 (60)	16 (80)	16 (80)
Comorbidities, *n* (%)			
0	8 (40)	10 (50)	6 (30)
1	6 (30)	10 (50)	11 (55)
2	5 (25)	0 (0)	3 (15)
≥3	1 (0.5)	0 (0)	0 (0)
Oral NSAIDs			
Currently use *n* (%)	2 (10)	3 (15)	5 (25)
Exercises: *n* (%)			
Currently use	10 (50)	4 (20)	8 (40)

Values are *n* (%) except where stated. % is percent of group

‘*group’* group acupuncture, *‘individual’* individual acupuncture, *‘standard’* standardised advice and exercise booklet only

### Clinical outcomes

No serious adverse events were reported. Minor adverse events (e.g. pain, bruising, symptom exacerbation) were reported by six participants during or after a total of 17/342 (4.9 % 95 % CI 2.6, 7.2) acupuncture treatments.

Raw scores for symptoms, as measured by the WOMAC, are shown in Table 2. Should a future fully powered trial be planned using the WOMAC pain score for the basis of a sample size calculation, the SDs for WOMAC pain at 14 weeks follow-up were 4.3 (‘group’) and 3.9 (‘standard’).

**Table 2:** WOMAC subscales and total

Arm	‘group’	‘individual’	‘standard’
Mean (SD), *n*			
Pain pre	9.5 (2.8), 20	10.9 (2.9), 20	9.3 (2.7), 20
Pain 14w	5.8 (4.3), 17	8.4 (2.8), 15	9.3 (3.9), 14
Stiffness pre	4.5 (1.4), 20	4.8 (1.7), 20	4.5 (1.2), 20
Stiffness 14w	3.1 (1.8), 17	3.4 (1.2), 15	4.0 (1.6), 14
Function pre	35.2 (9.6), 20	37.8 (8.4), 20	31.9 (9.3), 20
Function 14w	23.0 (16.1), 17	30.0 (10.1), 15	29.4 (11.5), 13
Total pre	49.1 (13.1), 20	53.4 (12.2), 20	45.6 (12.5), 20
Total 14w	31.9 (21.8), 17	41.8 (13.4), 15	42.8 (16.5), 13

Maximum scores for WOMAC are 20 (pain), 8 (stiffness), 68 (function), and 96 (total)

*Pre* baseline, *14w* 14 week follow-up, *‘group’* group acupuncture, *‘individual’* individual acupuncture, *‘standard’* standardised care only

Using an intention-to-treat analysis with complete case data only, the mean changes (95 % CI) in WOMAC pain scores for those providing follow-up data were ‘group’ (*n* = 17) −3.2 (−5.1, −1.4); ‘individual’ (*n* = 15) −2.4 (−4.1, −0.7); and ‘standard’ (*n* = 14) 0.4 (−1.4, 2.2). These changes were consistent with secondary outcomes including EQ-5D, OKS, numbers taking NSAIDs daily, troublesomeness, and global change, shown in Table 3.

**Table 3:** Other outcomes

Arm	‘group’	‘individual’	‘standard’
Mean (SD), *n*			
EQ-5D pre	0.545 (0.255), 19	0.480 (0.250), 20	0.555 (0.274), 19
EQ-5D 14w	0.639 (0.308), 17	0.660 (0.227), 14	0.560 (0.271), 13
OKS pre	22.8 (7.4), 20	22.4 (6.8), 20	24.2 (5.8), 20
OKS 14w	32.3 (11.1), 17	29.6 (7.7), 15	27.2 (7.7), 13
Using NSAID daily, *n*/*N*			
pre	11/20 (55 %)	8/20 (40 %)	10/20 (50 %)
14w	5/17 (29 %)	4/14 (29 %)	7/14 (50 %)
Troublesome^a^, *n*/*N*			
pre	7/20 (35 %)	9/20 (45 %)	5/20 (25 %)
14w	3/17 (18 %)	2/15 (13 %)	3/14 (21 %)
Global change^b^ *n*/*N*			
14w	7/17 (41 %)	9/15 (60 %)	2/14 (14 %)

*Pre* baseline, *14w* 14 week follow-up, *‘group’* group acupuncture, *‘individual’* individual acupuncture, *‘standard’* standardised care only, *NSAID* non-steroidal anti-inflammatory drug

^a^Highest two categories, extremely and very

^b^Highest two categories, much or moderately better

### Economic outcomes

Mean costs of acupuncture were £209 (SD £34) per person for group and £293 (SD £32) for individual acupuncture.

## Discussion

### Summary

Our database searches found that about 2 % of those registered with a GP are potentially eligible for a trial of acupuncture for knee OA. Recruitment was readily achieved within the target time and resources, but the overall follow-up rate (using one postal reminder only) was lower than desired. The piloted trial procedures were mostly satisfactory, and data completion rates were generally very good. Acupuncture in group format was acceptable to most participants, and clinical outcome data suggest that a future main trial is warranted.

### Strengths and limitations

The strengths of this pilot trial were success of recruitment, adherence to acupuncture and completion on schedule. We intended to recruit participants with severe symptoms, representing those suitable for consideration for arthroplasty, but our study sample is typical of other studies of conservative treatment for primary care patients with moderate pain and disability [31]. Group and individual acupuncture clinics were unavoidably held in different rooms, which could introduce bias, as for example one room was windowless. The engagement of a single acupuncturist who may not be typical is a limitation but minimised variability.

### Comparisons with existing literature

Another pilot study similarly identified 2 % of GP lists as potentially eligible for recruitment [32]. Recruitment to trials of acupuncture seems easier than to trials of NSAIDs [33].

Current evidence shows that acupuncture has a large effect size on knee pain compared with usual care [16] and the changes we identified, in comparison to a booklet control arm, are of the same order. Electrical stimulation seems important for acupuncture’s best effect in this condition [17] and [31]. The absence of relevant change in the standardised advice and exercise booklet care group is also consistent with the evidence of the clinically insignificant effect of standardised information leaflets for OA [24].

We are not able to comment on cost-effectiveness of acupuncture in this study: a previous study found that acupuncture for knee pain costing £314/person was cost-effective at £3889 per quality adjusted life year (QALY) [21]. Our acquisition costs of acupuncture showed smaller relative savings for groups compared to a previous study of supervised rehabilitation classes, which were £314/person individually and £125/person for group at 2003–2004 costs (£415 and £165, respectively, at 2014 costs) [34].

### Implications for definitive trial design

Our results suggest that research into group acupuncture’s possible role in the treatment gap in this patient population would be worthwhile. The current trial design was successful in most respects. The unacceptably low response rate to follow-up questionnaires in the standardised care arm needs addressing, for example by providing this group with additional personal contact as compensation for lack of contact with the acupuncture practitioner. For all participants, additional follow-up reminders with incentives and telephone or internet-based collection of minimum outcome dataset could improve follow-up.

Further questions arise, including whether the benefits of ‘group’ psychological aspects of group acupuncture could be optimised by manipulating the conversation [23] and whether delivery of acupuncture by less costly staff such as nurses [22] and improved clinic occupancy can reduce costs without any detrimental effect on potential effectiveness.

The choice of control group for studies of acupuncture for pain relevant to UK practice is increasingly challenging. By comparing acupuncture with no acupuncture, we aimed to address the everyday decision that faces patients and their GPs, though research recommendations in current NICE guidelines on OA prefer the comparison with sham acupuncture [24].

## Conclusions

We have demonstrated that a future trial testing the clinical and cost-effectiveness of the addition of a group acupuncture intervention to standardised advice to exercise for patients with knee pain attributable to OA, in primary care, is feasible. Participants are willing to be randomised, attend for treatment, and provide follow-up data. Further follow-up reminders, minimum data collection, and incentives should be considered to improve participant retention.

## Ethics approval and consent to participate

Written informed consent was obtained from all participants before enrolment. Ethics approval for the study was granted by NRES Committee South West—Cornwall and Plymouth, ref 11/SW/0277.

## Additional files

Additional file 1:
**ScrutiKnee participant information leaflet.** (PDF 207 kb)
Additional file 2:
**Advice and exercise leaflet for ScrutiKnee study.** (PDF 1436 kb)
Additional file 3:
**Acupuncture information leaflet for ScrutiKnee study.** (PDF 401 kb)
Additional file 4:
**ScrutiKnee acupuncture protocol for knee pain.** (PDF 58 kb)
Additional file 5:
**Other baseline and outcome questions for ScrutiKnee study.** (DOCX 37 kb)
Additional file 6:
**Responses to quantitative items in ScrutiKnee commentary.** (PDF 170 kb)

## Abbreviations

ACR: American College of Rheumatology
BMI: body mass index
CONSORT: consolidated standards of reporting trials
EA: electroacupuncture
EQ-5D: EuroQol five dimension quality of life instrument
GP: general practitioner
NICE: National Institute for Health and Care Excellence
NHS: National Health Service
NSAID: non-steroidal anti-inflammatory drug
OA: osteoarthritis
OKS: Oxford knee score
PenCTU: Peninsula clinical trials unit
QALY: quality adjusted life year
RCT: randomised controlled trial
UK: United Kingdom
UKCRC: UK Clinical Research Collaboration
WOMAC: Western Ontario & McMaster Index
