# Supplementary material for: Western medical acupuncture in a group setting for knee osteoarthritis: results of a pilot randomised controlled trial
Source: Pilot Feasibility Stud. 2016 Feb 16;2:10. doi: 10.1186/s40814-016-0051-5 (PMC5153913; doi:10.1186/s40814-016-0051-5)
Supplement: Additional file 1: — ScrutiKnee participant information leaflet. (PDF 207 kb) [file 40814_2016_51_MOESM1_ESM.pdf]

Participant Information Leaflet

SCRUTIKNEE
Scrutinising treatments for knee pain

We would like to invite you to take part in our research study. Before you decide whether to take part, we would like you to understand why the research is being done and what it would involve for you. This leaflet contains information about the study.

This is a study about non-drug treatments for pain from osteoarthritis of the knee. It is a 'pilot' study which will help us plan a larger study to find which treatment is best.

Part 1 of this leaflet tells you the purpose of this study and what will happen to you if you take part. Part 2 gives you more detailed information about the conduct of the study.

Talk to others about the study if you wish. Ask us if there is anything that is not clear.

Thank you for taking time to read this information

PART 1

What is the purpose of the research?

We want to find out whether or not painful osteoarthritis of the knee is helped by self-help advice and exercise, with or without the addition of acupuncture. We need to find out the best way to organise a large study, and how to recruit people - so we are running a small pilot study first, and it is this study that you are being invited to join. The results of this pilot study will be used to let us know how to develop and carry out a larger study involving more people.

What is the study about?

As we don't know which treatment is best, we need to find out by making comparisons between the different treatments. The three groups are i) advice and exercise, ii) advice and exercise and one-to-one acupuncture and

iii) advice and exercise and group acupuncture. If you volunteer to take part in the study you will not be able to choose which treatment you receive - it will be selected by chance using a computer programme. You therefore need to be happy to receive any of the three options.

Why have I been invited?
You have been invited to take part because you have osteoarthritis of the knee that causes moderate or severe pain and you have indicated your interest in the research. A total of 60 people will be included in the study.

Do I have to take part?
No. Participation is entirely voluntary. If you decide that you do not want to take part, simply tell the researcher when she telephones you, and the research team will not contact you again. If you decide not to join the study, this will not affect the standard of healthcare you receive.

What will happen to me if I take part?
You will be involved with the study for about 14 weeks and will need to attend the research centre in Plymouth once, at the start. At this visit, a research nurse will answer any questions you may have about the study and check that you are suitable to take part. If you are happy to take part you will be asked to sign a consent form.

The nurse will take some routine measurements of your height and weight and ask you a few more questions. You will also be asked to fill in some short questionnaires about your knee pain. The research nurse will use a computer programme to tell you whether or not you have been selected at random (like tossing a coin) to receive acupuncture, as an additional treatment to the advice and exercise booklet.

You will then be given an ‘advice and exercise’ booklet, containing exercises and self-management advice designed to help you manage your knee pain.

- This appointment will take about an hour.
- You are welcome to bring a friend or relative along with you if you wish.
- Please wear loose-fitting clothes to allow examination of your knees.

What will I be expected to do after that?
During the 14 weeks of the study you will be expected to follow the exercise programme as best you can, and try out the pain relieving techniques described in the booklet.

You will be asked to complete a form about your experiences of taking part in the study on several occasions over the course of the study and will be asked to repeat the questionnaires about your knee pain at the end of the study. You will be provided with reply-paid envelopes in which to return these forms.

So where does the acupuncture fit in?
As well as following the exercise programme, some people will be selected to receive acupuncture. This will be delivered in one of two ways, either on a one-to-one basis with just you and the therapist, or as part of a group where you will sit alongside other participants who are having the same treatment.

A course of between six and 10 treatments of acupuncture will be provided over twelve weeks, at the Cumberland Centre. The treatment will be given by a health professional (e.g. nurse or physiotherapist) employed within the NHS, who has a professionally recognised qualification in acupuncture.

How will having acupuncture affect my ability to donate blood?
If you plan to donate blood within six months of receiving acupuncture, you can request a letter to confirm that you have received acupuncture from a recognised professional.

What are the alternatives for treatment?
You are free to receive the usual care for your knee problem from your GP throughout the study, so you are not being denied any treatment. If you are taking medication for your pain, you may continue to do so. If you are taking medication for another condition, continue taking it as normal.

What are the possible disadvantages and risks of taking part?

- Acupuncture needles are very thin and rarely cause pain, though they quite often cause some aching. About 3 in 100 cause a little bleeding.

- A few people can feel nauseous or faint or experience a temporary increase in their pain either during or after acupuncture. Other responses

include feeling tired, relaxed or energised. If you do experience anything untoward you must let the therapist know.

- Research shows that acupuncture is a very safe treatment when given by properly trained clinicians, as in this study.

What are the possible benefits of taking part?
You will be receiving the treatment currently recommended by professional and national guidelines (NICE) for osteoarthritis – in the form of the advice and exercise booklet. Research shows that these can produce long-term benefit.
You may receive treatment with acupuncture as well as part of the study. It is not clear whether this provides any pain relief in addition to advice and exercise. Therefore, all participants will be helping in a study that may help other patients in the future (for example, showing whether acupuncture is worth providing for osteoarthritis of the knee in the NHS service or not).

Expenses and payments
We can refund your taxi or bus fare for the single visit to the research centre. However, if you are in the acupuncture group, we cannot refund you for travel to the clinic.

What happens after the research study stops?
You are advised to continue your advice and exercise programme as we know this is of benefit. If you have been chosen for a course of acupuncture, there are no facilities generally available for you to carry on with this treatment within the NHS.

What if there is a problem?
Any complaint about the way you have been dealt with during the study or any possible harm you might suffer will be addressed. The detailed information on this is given in Part 2.

Will my taking part in the study be kept confidential?
Yes. We will follow ethical and legal practice and all information about you will be handled in confidence. The details are included in Part 2.

This completes Part 1.

PART 2

Can you tell me more about the clinic visit?

At this appointment the research nurse will go through this information sheet with you and give you the opportunity to ask any questions you might have regarding the study. The nurse will then check that you are suitable for the study which includes a brief examination of your knee. If you are suitable and happy to take part you will be asked to sign a consent form, a copy of which will be given to you to keep.

The nurse will then question you about your medical history; measure your height and weight; and measure your pain threshold using a small hand held device, placed over a point near your knee and over a point in your arm. Pressure will be applied gradually and stopped as soon as you feel pain.

Finally, the nurse will ask you to fill out several short questionnaires about your knee pain and the impact this pain has on your daily life. This will take about 15 to 20 minutes. The majority of the questions only need you to choose and tick one box.

You will be given a form to take home, with questions and spaces for you to write any comments about your experience of taking part in the study. You will also be sent similar forms to fill in, up to three more times in the study.

If you have not been selected to receive the acupuncture, this is all you are asked to do. If you have been selected to receive acupuncture, the research nurse will tell you whether you have been allocated to individual or group treatment, and arrange for you to have an appointment at the Cumberland Centre in Plymouth. The research nurse will also give you an information leaflet which explains about acupuncture.

What about the questionnaires at the end of the study?

- You will be asked to fill out the questionnaires again after 14 weeks.
- The forms and questionnaires will be sent to you in the post, along with a pre-paid envelope for their return. If you forget to fill them in, a reminder will be sent to you. The research nurse will discuss how you prefer to receive this, for example text message, phone or email.

Does the research team need any other information from me?

- The staff at your surgery may be asked to inform the research team of the number of times you have seen your medical team, and the number of prescriptions you had, during in the study.
- At the end of the study, some people will be invited for interview about their experiences in the study. Further information will be provided about this at the time, and you will be given the opportunity to accept or decline.
- If you decide to leave the study before the end, you might also be contacted once by letter, to invite you to take part in an interview. If you choose not to respond, you will not be followed up any further.

If I am chosen for acupuncture what will I have to do?

On your first appointment you will be seen on your own by the acupuncturist who will be treating you. After you have answered some questions and given your consent, you will be given a short session of acupuncture (about 10 minutes). This is to test your likely response. For this session you will be treated resting on a couch. For all subsequent treatments you will be sitting in a chair.
The acupuncturist will then give you an appointment for the following week. For this appointment you will receive acupuncture in the selected way – either one-to-one with just you and the therapist present, or as part of a group with other people in the study. If you have acupuncture as part of a group, the group will be made up of both men and women with up to six people in each group having similar treatment.

You should wear comfortable loose fitting clothing that will allow the acupuncturist to treat your knees, without you having to undress.

- The treatment time will vary between 10 and 30 minutes, depending on your response.

- If your knee pain is no better after six treatments the acupuncture will be stopped.

- If you miss any treatment because you are ill or on holiday you can resume treatment at the next available appointment provided it is within 12 weeks of your first treatment.

What if relevant new information becomes available?
Sometimes we get new information about the treatment being studied. This is unlikely with advice, exercise and acupuncture, but if this happens, the research team will tell you and discuss it with you.

What will happen if I don't want to carry on with the study?
You are free to withdraw entirely from the study at any stage. We would like to offer you the chance to tell us the reasons why, but you do not have to do this. You are also free to withdraw from the treatments, in which case you may or may not be willing to be followed up in the usual way with questionnaires after 14 weeks.

What if there is a problem?
Complaints
If you have a concern about any aspect of this study, you should ask to speak to the researchers who will do their best to answer your questions [01752 764448]. If you remain unhappy and wish to complain formally, you can do this through the Plymouth NHS Trust Complaints Procedure. Details can be obtained from:

Patient Advice & Liaison Service (PALS)
Local Care Centre
Mount Gould
Plymouth PL4 7PY
0845 155 8121 or 01752 435204

Harm
In the event that something does go wrong and you are harmed during the research and this is due to someone's negligence then you may have grounds for a legal action for compensation against Plymouth (Teaching) NHS Trust but you may have to pay your legal costs. The normal National Health Service complaints mechanisms will be available to you.

Will my taking part in this study be kept confidential?
Any personal information that we collect about you, and any consent forms that you complete, will be stored securely in locked filing cabinets at the Peninsula Medical School and will only be used for the purposes of the research. Your name and address will be stored securely on a computer, accessible only by members of the study team for the purpose of contacting you, and stored separately from the research information collected in the study. Your name and address will not appear on any study forms or questionnaires so that you cannot be recognised from them.

You will be given a unique identification code for administration purposes. When the information is analysed your name and address will be removed so you cannot be identified from it. Only anonymised data will be stored on computer. You have the right to check the accuracy of data held about you and to correct any errors. Procedures for the handling, processing, storage and destruction of your data will be compliant with the Data Protection Act 1998.

Some parts of the data collected for the study may be looked at by authorised representatives of regulatory authorities to check that the study is being carried out correctly. Should this occur, all such individuals will have a duty of confidentiality to you as a research participant. Your confidentiality can only be breached in exceptional circumstances, for example if the research team believe your or another person’s health is seriously at risk.

Involvement of your General Practitioner

Your GP will be notified of your participation in the study, if you consent.

What will happen to the results of the research study?

We will produce a short summary of the study findings that can be sent to participants who request it. We also hope to publish the results of this study in journals that will be read by other healthcare professionals. You will be able to obtain a copy by writing to us.

Who is organising and funding the study?

The study is led by Dr Adrian White, Clinical Research Fellow in Primary Care at the Peninsula Medical School, supported by the Peninsula Clinical Trials Unit. The study will be sponsored (overseen) by the Plymouth (Teaching) PCT. The study is funded by a research grant awarded by the National Institute of Health Research (NIHR) Research for Patient Benefit scheme. Nobody will receive any payment for your participation in this study.

Who has reviewed this study?

All research in the NHS is looked at by an independent group of people, called a Research Ethics Committee, to protect your interests. The study has been reviewed and given a favourable opinion by Cornwall and Plymouth Research Ethics Committee.

Further information and contact details

If you require further information about this study, please contact the study coordinator, Liz Tough, on 01752 764448. If you are undecided about taking part, your own GP or nurse may be able to advise you. If you need general information about medical research, please ask the study coordinator for a leaflet about taking part in clinical trials.
