# Supplementary material for: Western medical acupuncture in a group setting for knee osteoarthritis: results of a pilot randomised controlled trial
Source: Pilot Feasibility Stud. 2016 Feb 16;2:10. doi: 10.1186/s40814-016-0051-5 (PMC5153913; doi:10.1186/s40814-016-0051-5)
Supplement: Additional file 2: — Advice and exercise leaflet for ScrutiKnee study. (PDF 1436 kb) [file 40814_2016_51_MOESM2_ESM.pdf]

PENINSULA
MEDICAL SCHOOL
UNIVERSITIES OF EXETER & PLYMOUTH

NHS
Plymouth

ScrutiKnee
Scrutinising treatment for knee pain

ScrutiKnee

Advice and Exercise Booklet

This booklet contains advice, information and exercises to help you manage your knee pain

Advice and Exercise Booklet

Contents

What is osteoarthritis?....................................3

Why have I got osteoarthritis?........................3

Are my joints wearing out?..............................4

Why do I get pain?..4

How does osteoarthritis affect the knee?.......5

Exercise 1 Leg swings......................................10

Exercise 2 Heel slides......................................11

Exercise 3 Muscle squeeze..............................13

Exercise 4 Leg raise from bent........................14

Exercise 5 Straight leg raise............................16

Exercise 6 Sit to stand.....................................18

Exercise Summary...20

Osteoarthritis of the knee

What is osteoarthritis?

Osteoarthritis is an ailment that affects the joints. 'Osteo' means bone and 'arthritis' means joint damage and swelling (inflammation). Most of the joints of the body are covered with cartilage - a thin, shiny layer which allows the joint to move smoothly and without pain. In osteoarthritis this cartilage becomes damaged, usually in the part of the joint that carries the most load. The bone underneath the cartilage responds to this damage by becoming thicker. Sometimes the bone at the edges of the joint can grow outwards forming bony spurs which are called osteophytes. These can be clearly seen on X-ray.

The knee joint is surrounded by a tough fibrous 'sleeve' called the joint capsule. The capsule has an inner lining called the synovium which produces fluid. Normally this fluid lubricates the joint and allows the joint to move smoothly. In osteoarthritis the synovium becomes thickened. Extra fluid is produced and the joint can swell.

Why have I got osteoarthritis?

We don't really know why some people develop osteoarthritis and other people don't.

However, we know that the chance of developing arthritis increases with age (although developing arthritis isn't an inevitable part of growing old). We also know that arthritis is more likely to affect people who are obese or overweight, or who have had a previous injury or accident affecting the joint (e.g. whilst playing sport or after a fall).

Are my joints wearing out?

No. The whole process of osteoarthritis is one of adaption, and as such it is better described as ***'wear and repair'*** rather than *'wear and tear'*. The development of osteoarthritis usually takes place over a number of years. However, occasionally it can develop rapidly, and the pain and loss of movement can become severe quite quickly.

Why do I get pain?

Interestingly the joint cartilage, like your hair and nails, has no nerve supply so damaged cartilage cannot in itself produce pain. Pain comes from the now unprotected bone which is under the damaged cartilage, and from the ligaments and capsule which surround the joint and become stretched when the joint is swollen.

"Osteoarthritis of the knee affects different people in different ways. Some people have a problem with only one knee, others with both knees. Pain is the main problem for some people while others find their main problem is difficulty in walking".
Research shows that about one third of people with osteoarthritis get worse over time. However, it also shows that about one third of people stay the same, and one third get better.
"Therefore, it is not helpful to compare the experiences of one person with another, and the eventual outcome for any one individual with osteoarthritis cannot be predicted".

Arthritis Research UK
Providing answers today and tomorrow

How does osteoarthritis affect the knee?

Osteoarthritis usually affects the inner half of the knee joint and the back of the knee cap. If the knee cap is affected, a crunching and grinding sensation can be felt (and often heard) especially when going up and down stairs.

Spending too long with pressure through the joint, either by standing or walking, may cause the joint to swell. If the swelling stops you bending your knee as far as you can normally, it is best to ease off walking for a couple of days to allow the joint to settle.

Managing your pain

While there is currently no cure for osteoarthritis, there are a number of things you can do to help pain, stiffness and swelling.

1. Keep your joints moving. You need to strike a balance between too much activity and too much rest. Most people find that if they sit still for too long they stiffen up, making it harder to get going again.
Try not to sit with your knees bent in one position for longer than 15 to 20 minutes.

Managing your pain

1. Exercise and rest – strike the right balance

2. Maintain a healthy weight

3. Keep your leg muscles strong

4. Use painkillers to help you exercise

5. Use warmth or ice

6. Use a walking stick and wear cushioned shoes

2. Do not put a pillow under your knee at night. Whilst this may ease your pain, if you do this regularly it can cause the muscles at the back of your knee to shorten, leaving your knee permanently bent. Even if the pain is severe always try and straighten your knee as far as you can a few times during the day.

3. Pace your activities. For most people the best advice is 'little and often'. For example, do house work or gardening in short spells interrupted by short spells of rest. You may find that your knee pain gets worse after an activity. If this pain lasts for an hour or two but then settles again, that is okay. If the pain continues for the rest of the day, then you have done too much. Even if an activity does cause extra pain ***it does not mean that you have damaged the joint any further***. So don't worry if from time to time you misjudge how much you can do.

4. Things that could help your pain. Make use of pain relieving tablets or creams that you have been prescribed by your GP or you have bought yourself. Applying heat to the knee can also be helpful. Try using a microwavable wheat bag or hot water bottle wrapped in a towel, but take care not to burn yourself. Some people find a bag of frozen peas, wrapped in a damp tea towel (to prevent an ice burn) helps more. Keep the heat or ice pack on for up to 20 minutes.

Don't use heat or ice if there are patches of skin around your knee where you can't feel hot or cold i.e. **areas of numbness.**

5. Reduce the load. When you are out walking **wear cushioned (training) shoes**. These act as shock absorbers for your knees. If your knees are particularly painful **walk using a stick or sticks.** If one knee is painful, hold the stick in the hand opposite to the painful knee.

6. Use a hand rail for support when climbing stairs.
If your knee is particularly painful go upstairs one at a time with your least painful or pain free leg first. Come downstairs with your most painful leg first, using the rail for balance and support. As the muscles in your leg get stronger – by doing the exercises listed in this booklet - going up and down stairs should become easier and less painful.

Research shows that being obese or overweight can increase a person's risk of developing osteoarthritis in the knees. Gaining weight or staying obese increases the chance of the osteoarthritis becoming progressively worse. Try and maintain a healthy weight. Losing even a few pounds can help.

Exercises

Guidelines on exercising

These exercises are designed to help you move and strengthen your knee without putting pressure through the joint. It is important that your knee pain does not get worse when you are exercising. **It is NOT a case of 'no pain no gain'**.

However, when you do the exercises you may feel aching in the muscles. That is okay. This aching is simply an indication that the muscles are weak and they are becoming tired.

Whilst it is okay to continue exercising if the muscles are aching, it is not advised to exercise once the muscles are completely fatigued. A muscle is fatigued when it starts to shake during exercise, or when it becomes increasingly difficult to hold the muscle tight or control movement, such as in the straight leg raise exercise (see later).

Helping the pain

You may find it easier to exercise if you take some painkillers beforehand or you apply heat or ice to your knee – whichever helps your pain the most.

How often and how many?

Whilst the exercises listed below come with advice on how many and how often, this is just a guide. Aim towards this number but be guided by how your muscles and knee joint feels, and if you can't do them all at first, build up to this number gradually.

You may find that the amount of exercise you can do will vary from day to day. This is normal, so don't worry. **'Little and often' is better than 'not at all' or 'all at once'.**

The exercises are listed in order of difficulty. The first two exercises are designed to get the joint moving and warm up the muscles.

The following four exercises are designed to strengthen the muscles around your knee. **Strengthening the large muscles of your leg helps protect the joint.**

Exercise is a treatment recommended by the National Institute of Health and Clinical Excellence (NICE) in the UK, for all people with arthritis, irrespective of age, pain severity and disability.

When exercising

1. Be guided by how the knee and muscles feel
2. Little and often is better than not at all or all at once
3. Strengthen the muscles around your knee to support the joint and make it feel more stable
4. Exercising in water can help, but swimming breast stroke can put strain on the knees

Exercise 1 Leg swings

Purpose

This exercise is **useful if your knee is feeling a bit stiff and achy**. It is also useful as a warm up exercise for the strengthening exercises or before you start any strenuous activity, such as gardening or going for a walk.

Starting position

Sit on the edge of a bed, bench or chair so that your feet aren't touching the ground.

Action

Gently swing your legs, either alternately or both together - whichever you find most comfortable. Keep your legs relaxed. You are not aiming to straighten your knees out fully with this exercise.

Comments

Aim to do this exercise for up to 5 minutes, two to three times a day or whenever your knees are feeling stiff or achy.

1. Swing legs alternately

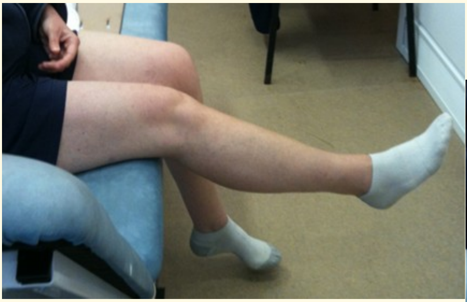

2. Swing both legs together

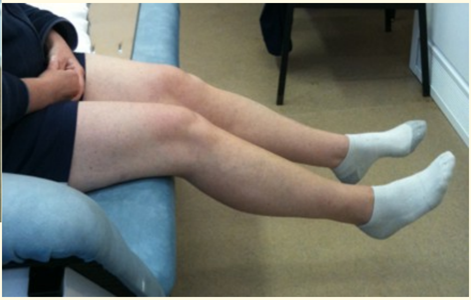

Exercise 2 Heel slides

ScrutiKnee

Purpose

If you can't find anywhere to sit that is high enough to allow you to swing your legs then try this exercise instead. If you can do the leg swing exercise do this exercise as well as it moves the joint in a slightly different way. The purpose of this
exercise is to **move the joint through its fullest range, both bending and straightening.**

Starting position

Lie in a comfortable position, either on your bed or sofa (whichever is firmest) in a way that allows you to bend your knee without putting strain on your back or hips. Have your other leg either bent or straight, whichever is more comfortable.

Equipment needed

You will need something shiny or slippy to place under your leg e.g. a tray, piece of lino or polished wooden board. The slippy surface will make it easier for you to bend your knee up and down.

Action

Slide your heel towards you along the board until you feel your knee tighten. Then try and bend it a little more before slowly, sliding your heel away from you until your knee is as flat as you can get it.

You may find that as you bend your knee up, your leg will want to fall out to the side. This will happen if your thigh and bottom muscles are weak. Try and keep your knee in line with your hip and your heel, so you are sliding your leg up and down in a straight line.

Comments

Aim to slide your leg up and down like this for up to 5 minutes. If you can, do this two to three times a day or whenever your knee is feeling stiff or achy.

1. Start and finish position

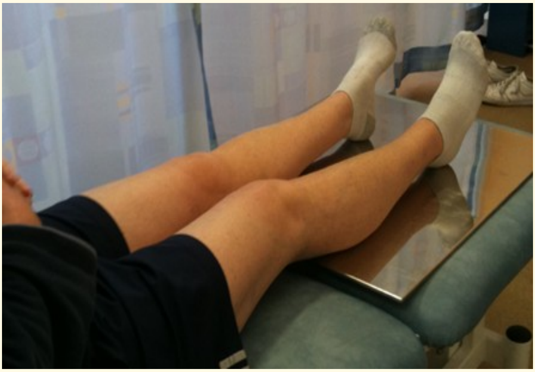

2. Slide up with leg in straight line

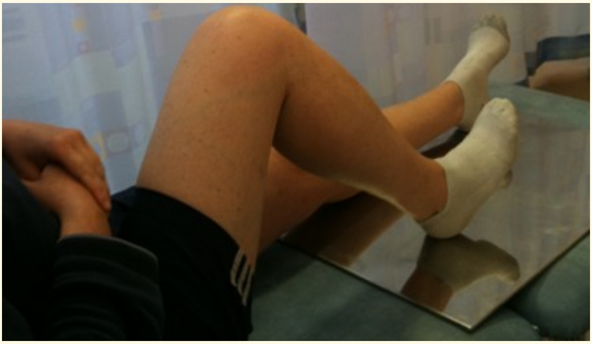

Exercise 3 Muscle squeeze

ScrutiKnee

Purpose

This exercise can be done at any time throughout the day. For example, while you are sitting eating breakfast, reading the paper or watching television. The purpose of this exercise is **to strengthen the large muscles that help support your knee** (i.e. those in your bottom and thigh).

Strong muscles reduce the pressure or load which passes down through your knees when you are standing or walking.

Starting position

Sit in a chair making sure that your feet are flat on the floor, and your feet are about a foots length distance apart.

Equipment needed

You will need a large cushion, pillow or child's football.

Action

Place the pillow between your knees. Now squeeze as hard as you can, tightening your thigh muscles and clenching your bottom muscles tight. Hold for 5 seconds, then gradually release.

Comments

You can do as many of these as you like. This exercise is designed to be done **little and often** - guided by the feeling of fatigue in the muscles.

Modifications

If you get pain around your knee cap, try doing the exercise with your knees a bit straighter. As your muscles get stronger the pain around your knee cap may ease, allowing you to exercise with your knees bent more (e.g. at right angles).

1. Squeeze knees at right angles

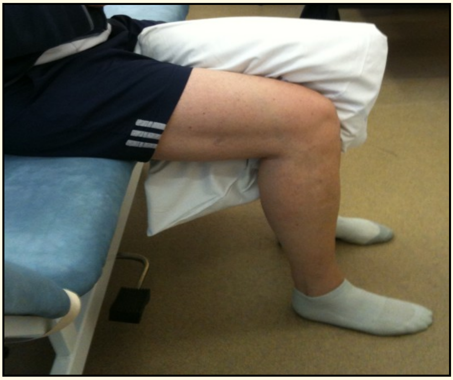

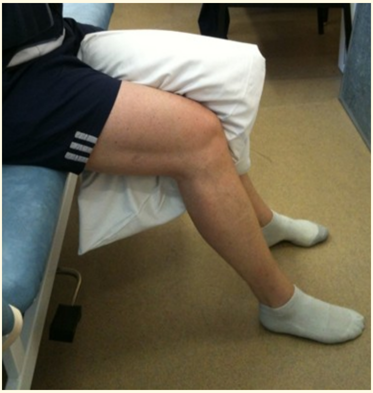

2. Modification with straight knees straighter

Exercise 4 Leg raise from bent

Purpose

This exercise **builds up the strength in your quadriceps - the large muscle at the front of your thigh which helps support your knee.** This exercise can be done as a warm up for the next exercise (straight leg raise), or as an alternative if your muscles are too weak, or if the knee is too painful to do the straight leg raise.

Starting position

Lie propped up on your bed.

Equipment needed

You will need a bath towel and a wine or squash bottle. Wrap the bottle in the towel.

Action

Place the roll under your knee so that your knee is bent at around 45 degrees. Point your toes to the ceiling, stretching the back of your calf, and then raise your heel off the bed straightening your knee as much as you can. Hold your leg in this position for 5 seconds and then slowly lower.

You should feel the muscles at the front of your thigh working quite hard and you may feel a stretch at the back of your knee, which is normal.

Comments

Repeat the exercise 10 times, holding each for 5 seconds. Have a minutes rest then repeat 10 more times. Rest again for a minute then do 10 more. So 30 raises in all. Aim to do this exercise twice a day.

If you have painful arthritis in both knees, you will want to exercise both legs, so exercise one leg while you rest the other.

Modifications

After exercising for a week or two you may find this exercise becomes too easy. To make it harder, you can **add a small weight (2-4lbs)** around your ankle**.** You can buy strap on weights from a sports shop or instead fill a small bag, pair of socks or stockings with something like sand or rice and balance this over your ankle.

1. Start position

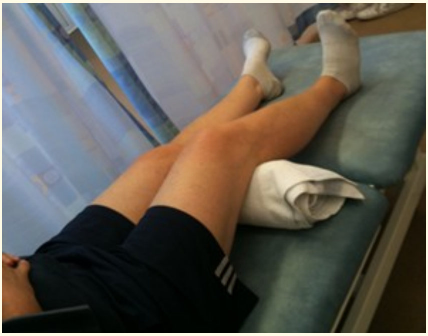

2. Raise

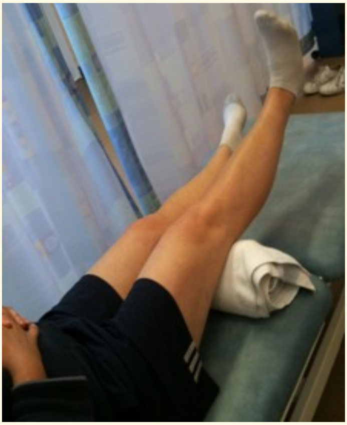

3. Weight added

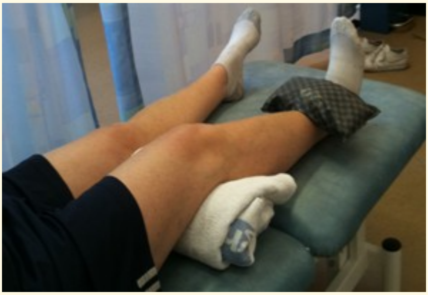

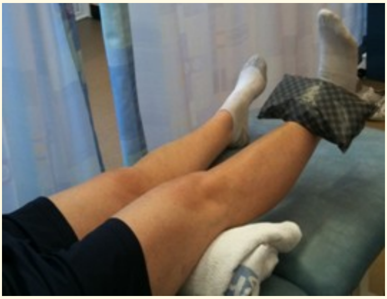

Exercise 5 Straight leg raise

Purpose

This is the most strenuous exercise but it is also **the most effective way of strengthening the quadriceps muscle**. If you can raise your leg with your knee straight and without pain then you should do this exercise.

Starting position

Position yourself on a bed or sofa. The exercise is easier and puts less stress on your back and hips if you lay flat rather than sitting upright – but select a position that feels most comfortable for you.

Action

As with the previous exercise, start by pointing your toes up to the ceiling stretching the back of your knee and your calf muscle.

Push your knee as straight as you can, then raise your leg only about 6 inches. Hold your leg straight and raised for 5 seconds, then slowly lower. You will feel the muscles at the front of your thigh working hard to hold your leg straight.

Comment

This is an exercise that can make the muscles ache and can cause the muscle to shake with fatigue. Remember, **don't exercise through fatigue but a little muscle ache is okay.** As with the 'leg raise from bent', aim to do a total of 30 raises split into three groups of 10, with a minutes rest between each set of 10. You may not be able to do the number of repetitions recommended straight away as your muscles may be quite weak. If this is the case, persevere.

Gradually increase the number of repetitions each week, until you reach the recommended number.

1. Start position

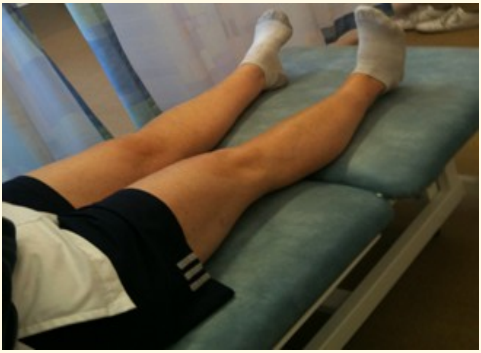

2. Straight leg raise

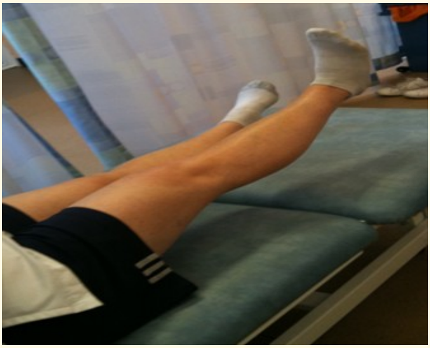

Exercise 6 Sit to stand

Purpose

This exercise builds up the strength in your quadriceps (thigh) and the gluteal (buttock) muscles - the muscles that help support your knee. **This exercise also helps improve balance and co-ordination.**

Starting position

Sit in a dining chair making sure that your feet are flat on the floor, and your feet are about a foots length distance apart.

Action

With equal weight through both legs, push yourself up from the chair **slowly** straightening your knees. Try not to let your knees collapse inwards during this movement. Once you are upright and balanced, **slowly** lower yourself back down into the chair. To exercise the muscles properly it is essential that you do this exercise slowly and that you control the knee bending and straightening movement.

Comments

Aim to do up to 10 repetitions at a time (pushing up and then lowering down counting as one repetition), twice a day.

Modifications

If you have pain behind your knee cap or if your leg muscles are weak, you may find this exercise difficult. This exercise should not be painful. If it is, try using your arms to help push yourself up and then lower yourself back down into the chair. As your leg muscles get stronger use your arms less.

If you can't do this exercise without pain, don't worry. You can either leave the exercise out altogether, or you may want to try it again once your leg muscles have been strengthened by the other exercises.

And finally…

- During the day, whether standing or sitting get into the habit of clenching and releasing your bottom muscles (you should feel your thigh muscles tightening at the same time). This will help the muscles get stronger.
- People often ask if swimming is a good exercise. Exercising in water can be helpful as the support from the water can ease the pressure on the joints. The buoyancy of the water should allow you to move more freely and with less pain. However, swimming breast stroke is generally not recommended as the screw kick action can strain the knees. Using a straight leg kick or swimming gentle side stroke is often better.

These are exercises for life. As your knee pain improves the temptation is to stop exercising.

All six exercises help maintain the movement and strength in your leg/s. If you stop exercising the strength will be lost and as a result your knee pain may return or get worse. It is advised that you do these exercises every day for 3 months. After this time, exercising three times a week rather than every day should be sufficient to maintain any improvement.

Exercise Summary

Exercise	Purpose	How often	How long for
**1. Leg swings**	Ease stiff and achy joints.<br><br>Warm up for strengthening exercises.	Little and often throughout the day.	Up to 5 minutes at a time.
**2. Heel slides**	To maintain and improve knee movement.<br><br>Warm up for strengthening exercises.	Twice a day e.g. once after breakfast and once after your evening meal.	Up to 5 minutes at a time.
**3. Muscle squeeze**	To strengthen the thigh and buttock muscles that support your knee.	Little and often throughout the day e.g. when sitting having a meal, reading a book or watching TV.	Hold each squeeze for 5 seconds then slowly release. Up to 20 repetitions in one go.

**4. Leg raise from bent**	To strengthen the thigh muscles that support your knee.<br><br>An alternative to the *straight leg raise* if your muscles are weak.	Twice a day e.g. once after breakfast and once after your evening meal.	Hold each raise for 5 seconds. Aim to do 30 raises in each session. If your muscles are weak, gradually build up to 30 raises over 3 to 4 weeks.
**5. Straight leg raise**	The quickest way to strengthen the thigh Muscles.<br><br>An alternative to the *leg raise from bent*, if you have pain behind the knee cap when you straighten your knee.	Twice a day as above.	Hold each raise for 5 seconds. Aim to do 30 raises, split into 3 groups of 10, with a minutes rest between each set of 10. If your muscles are weak, gradually build up to 30 raises over 3 to 4 weeks.
**6. Sit to stand**	To strengthen the thigh muscles that support your knee.<br><br>To improve balance and co-ordination.	Twice a day as above, but each time you get in or out of a chair, try to control the movement.	Up to 10 repetitions making sure the movement is slow and controlled.

This booklet has been compiled by Chartered Physiotherapist, Dr Liz Tough in consultation with expert physiotherapists and patient representatives. Information and pain management advice has been adapted from Arthritis Research UK Information booklets. www.arthritisresearchuk.org.

Available in larger text on request

Acknowledgements: Members of ScrutiKnee patient representative advisory group. Expert physiotherapists: Linda Knott Clinical Specialist Pain Management Torbay Hospital; Dr Nadine Foster Professor of Musculoskeletal Health in Primary Care Arthritis Research, Keele University. Victoria Eyre. Research Trial Coordinator PenCTU.

PENINSULA MEDICAL SCHOOL UNIVERSITIES OF EXETER & PLYMOUTH

ScrutiKnee

NHS Plymouth
