# Supplementary material for: Western medical acupuncture in a group setting for knee osteoarthritis: results of a pilot randomised controlled trial
Source: Pilot Feasibility Stud. 2016 Feb 16;2:10. doi: 10.1186/s40814-016-0051-5 (PMC5153913; doi:10.1186/s40814-016-0051-5)
Supplement: Additional file 3: — Acupuncture information leaflet for ScrutiKnee study. (PDF 401 kb) [file 40814_2016_51_MOESM3_ESM.pdf]

Acupuncture Information leaflet

SCRUTIKNEE

What is acupuncture?

Acupuncture is an alternative medicine that involves inserting very thin needles through the skin and into the muscle beneath (see figure 1). The needles are manipulated by hand, by gently rotating them clockwise and anti-clockwise within the muscle. The manipulation stimulates the release of the body's natural pain killers (e.g. endorphins and serotonin) which can help reduce pain.

Figure 1.

Acupuncture needles around the knee

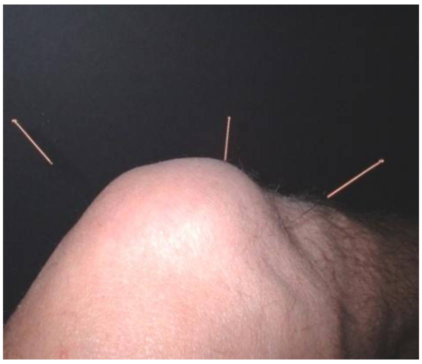

The acupuncture treatment will be given by a fully qualified and registered acupuncturist at the Cumberland centre in Plymouth.

Acupuncture does not interfere with other treatments for your knee, so please continue your painkillers and exercises in the normal way.

Acupuncture does not interfere with other treatments for your knee, so please continue your painkillers and exercises in the normal way.

What will the acupuncture treatment involve?

On your first appointment you will be seen on your own.

You should wear comfortable loose fitting clothing that will allow the acupuncturist to treat your knees, without you having to undress.

During this appointment:

- The acupuncturist will take a brief medical history so that they are aware of any health issues that may affect your response to the treatment. For example, if you are diabetic or have low blood pressure, there is an increased chance that you may feel faint during the treatment.
- You will be invited to sign a ‘consent to treatment’ form. This is part of the Primary Care Trust policy on needle and injection therapies.
- The acupuncturist will ask you to record the level of pain you have experienced over the past week using a 0 to 10 scale. 0 represents no pain, and 10 represents the worst pain you can imagine. You will be asked to record your level of pain again after your sixth session of acupuncture.

This first treatment with acupuncture will test your likely response and will last around 10 minutes. You will be treated resting on a couch in case the acupuncture makes you feel faint or light headed, and you need to be laid flat. The acupuncturist will insert between four and eight needles into the muscles above and below your knee (see figure 1). If both knees are painful, both knees can be treated if you wish.

Sometimes acupuncture can make you feel drowsy. If you do feel like this and if you are driving home, you will be advised to sit and wait until you feel alert enough to drive.

What should I expect to feel during the acupuncture?

People usually don’t feel anything when the acupuncture needles are inserted. However, you can occasionally feel a sharp pinprick sensation. If this is the case and the sensation persists, let the acupuncturist know. They will remove the needle and then reinsert it in a slightly different place so that it isn’t painful.

Once all the needles are in place, the acupuncturist will ask you whether or not you feel any sensation around the needles. The sensations you may feel include: a dull ache, heavy, tingling, a bee sting sensation, or a feeling of warmth.

- The acupuncture treatments will be carried out with you sitting in a chair.
- You can bring a book to read whilst you are having your treatment.
- You should not have acupuncture on an empty stomach as it could make you feel faint or light headed.

The strength of the acupuncture you receive will depend on your response to the previous treatment – that is whether your pain is better, the same, or worse. The strength of the acupuncture can be increased by adding more needles (up to eight), keeping the needles in for longer (up to 30 minutes), or stimulating the needles more vigorously, either by hand or by using electrical stimulation. For electrical stimulation, a small electrical current is passed through the needles using a battery powered electro-acupuncture unit, similar to a TENS machine (see figure 2). This produces a buzzing, tingling sensation at the needles, and can sometimes cause the muscles to twitch

<u>Figure 2.</u> Electro-acupuncture to the knee

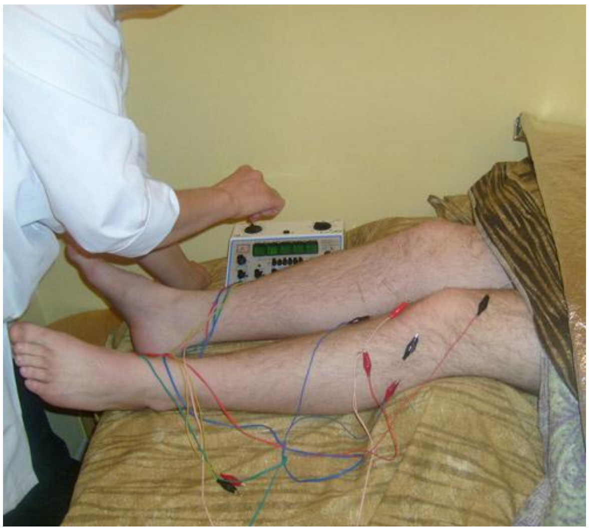

How many treatments will I have?

The basic course is six sessions, with a further four if you are getting better. This is judged from your pain score.

Sessions are given once a week for the first eight, then fortnightly. No treatment will be given after 12 weeks.

If you miss any treatment because you are ill or on holiday you can resume treatment at the next available appointment, provided it is within 12 weeks of your first treatment.

What are the risks?

- You may feel nauseous or faint or experience a temporary increase in pain either during or after acupuncture. Other more common responses include feeling tired, relaxed or energised. If you do experience anything untoward you must let the acupuncturist know.
- Needles may cause a little bleeding and associated bruising, however this is very rare (roughly 3 in 100 needles).
- Research shows that acupuncture is a very safe treatment when given by properly trained clinicians. The clinician delivering the acupuncture in this study is a specialist, with a professionally recognised qualification in acupuncture.
- There is no risk of infection. All the needles are sterile single-use disposable needles. All usual precautions regarding hygiene will be taken as required by professional guidelines.

What if there is a problem?

Please see the full details in your Participant Information Leaflet.

In the event that something does go wrong and you are harmed during the research and this is due to someone's negligence then you may have grounds for a legal action for compensation against Plymouth (Teaching) NHS Trust but you may have to pay your legal costs. The normal National Health Service complaints mechanisms will be available to you. Details can be obtained from:

Patient Advice & Liaison Service (PALS)
Local Care Centre
Mount Gould
Plymouth
PL4 7PY
0845 155 8121 or 01752 435204

Thank you for your participation in this study.
