# Supplementary material for: Western medical acupuncture in a group setting for knee osteoarthritis: results of a pilot randomised controlled trial
Source: Pilot Feasibility Stud. 2016 Feb 16;2:10. doi: 10.1186/s40814-016-0051-5 (PMC5153913; doi:10.1186/s40814-016-0051-5)
Supplement: Additional file 4: — ScrutiKnee acupuncture protocol for knee pain. (PDF 58 kb) [file 40814_2016_51_MOESM4_ESM.pdf]

ScrutiKnee Acupuncture Protocol

Acupuncture will be provided according to a protocol with some fixed and some individual points; the protocol has been developed from published studies and commented on and approved by senior members of the British Medical Acupuncture Society (BMAS). Acupuncture will be delivered using a Western, scientific approach, i.e. that the needles stimulate peripheral nerves in a particular way to achieve desired symptom relief through changes to the central nervous system. The study acupuncture will be delivered by a fully trained and registered acupuncturist who is a member of the Acupuncture Association of Chartered Physiotherapists.

Acupuncture treatment protocol

Treat both knees, if affected.

Patient posture

On couch (semi-supine for comfort) for first and second treatments, then may sit for all other treatments unless history of fainting with acupuncture.

Treatment schedule

Six treatments at weekly intervals, then reassess. If no response, discharge; if response, continue weekly for two weeks (i.e. 8 treatments in 8 weeks); then reduce to fortnightly. Total 10 treatments in 12 weeks.

Points

SP9, SP10, ST33*, ST36; and up to four other local points chosen for tenderness and the site of pain
Standard needles and insertion technique
Needle length as appropriate: 30 mm adequate for initial treatment and for small build; all other cases, 40/60 mm. Needle diameter 0.25 mm.
Insert needles down to the muscle layer or fascial plane and then a further 10/20 mm into deep layer.
If persistent sharp pain occurs with any needling, remove that needle; reinsert when sharp pain gone.

Level of stimulation

Manual stimulation means rotation (with lift and thrust if necessary) to elicit *de qi* if possible. Electrical stimulation means increase intensity until causes muscle contractions or is as strong as comfortable.
Over the course of treatment, titrate strength of stimulation upwards (no stimulation > manual stimulation > electroacupuncture) until the patient shows a response, then continue that level.
Treatment 1, ‘test’
Do not stimulate needles; remove after 5 minutes. If patient feels faint, remove all needles and lie patient supine; repeat same initial treatment on couch next week.
Treatment 2
(If good pain relief from the above, simply repeat the same but with 10 minute needle retention)
If no response, manual stimulation and Retain needle for 20 minutes.
Treatment 3 onwards

If no response, apply EA alternating 2/80 Hz between ST33–ST36 & SP9–SP10 for 30 minutes. Good strong sensation or muscle contraction (whichever first), but not painful.

*Note: ST33 is chosen instead of ST34 as needle more certain to enter vastus lateralis.

This protocol is derived from five recent large clinical trials which in turn developed protocols from the literature or from consensus of acupuncture practitioners or both, revised by taking into consideration neurophysiological principles (Mike Cummings, Medical Director BMAS), and by the need for standardisation and practicality of application in a group acupuncture setting, by healthcare staff with a varied range of acupuncture experience.

Equipment
Acupuncture needles used in this study will be CE marked, manufactured by Seirin
Electroacupuncture apparatus to be AS Super 4.
All needles single-use disposable and after use will be disposed of in yellow box provided in clinic. Cotton wool swabs to be disposed of with clinical waste. All materials to be ordered by study coordinator.

Compliance
The acupuncturist will record each attendance with the date.
