# Supplementary material for: Western medical acupuncture in a group setting for knee osteoarthritis: results of a pilot randomised controlled trial
Source: Pilot Feasibility Stud. 2016 Feb 16;2:10. doi: 10.1186/s40814-016-0051-5 (PMC5153913; doi:10.1186/s40814-016-0051-5)
Supplement: Additional file 5: — Other baseline and outcome questions for ScrutiKnee study. (DOCX 37 kb) [file 40814_2016_51_MOESM5_ESM.docx]

**BASELINE**

**1. Have you been advised (for example, by your GP, nurse or physiotherapist) to do some exercises for your knee pain?**

| Yes | **🗌** | | | No | | | **🗌** | | |  |  |
| --- | --- | --- | --- | --- | --- | --- | --- | --- | --- | --- | --- |
| \| If Yes, how much did these exercises reduce your pain? \| \| \| \| \| \| \| \| \| \| \| \| \| --- \| --- \| --- \| --- \| --- \| --- \| --- \| --- \| --- \| --- \| --- \| --- \| \| Not at all \|  \| A little bit \|  \| Moderately \|  \| Greatly \| \| \|  \| Totally \|  \| \|  \| \| \| \| \| \| \| \| \| \| \| \| \| **🗌** \|  \| **🗌** \|  \| **🗌** \|  \| \| **🗌** \|  \| \| **🗌** \|  \|   **2. Over the past 7 days, how often did you take a walk outside your home or garden for any reason, for example for fun or exercise, walking to work, walking the dog or other similar activities?** | | | | | | | | | | | |
| Never | |  | Seldom (1-2 days) | |  | Sometimes (3-4 days) | |  | Often  (5-7 days) | |  |
| **🗌** | |  | **🗌** | |  | **🗌** | |  | **🗌** | |  |

| **🡻** |  | |  | | **🡻** | | | | |  | | **🡻** | | | | | |  | | **🡻** | | | | |  | |  |  |  |  |
| --- | --- | --- | --- | --- | --- | --- | --- | --- | --- | --- | --- | --- | --- | --- | --- | --- | --- | --- | --- | --- | --- | --- | --- | --- | --- | --- | --- | --- | --- | --- |
| Please go to next question |  | **2a On average, how many hours per day did you spend on these activities on these days?** | | | | | | | | | | | | | | | | | | | | | | | | | | | | |
|  |  | Less than 1 hour | | | | |  | | 1-2  hours | | | | | |  | | 2-4 hours | | | | |  | | More than 4 hours | | | |  | | |
|  |  | **🗌** | | | |  | | **🗌** | | | | | |  | | **🗌** | | | | | |  | **🗌** | | | | | | |  |
| **3. Over the past 7 days, how often did you engage in light sport or recreational activities such as ‘light’ cycling on an exercise bike, bowls, yoga, tai chi or other similar activities?** | | | | | | | | | | | | | | | | | | | | | | | | | | | | |  |  |
| Never |  | | | Seldom (1-2 days) | | | | | | |  | | Sometimes (3-4 days) | | | | | |  | | Often (5-7 days) | | | | |  | | |  |  |
| **🗌** |  | | | **🗌** | | | | | | |  | | **🗌** | | | | | |  | | **🗌** | | | | |  | | |  |  |

| **🡻** |  | |  | **🡻** | | | | |  | **🡻** | | | | |  | **🡻** | | | |  |  |  |  |
| --- | --- | --- | --- | --- | --- | --- | --- | --- | --- | --- | --- | --- | --- | --- | --- | --- | --- | --- | --- | --- | --- | --- | --- |
| Please go to next question |  | **3a On average, how many hours per day did you spend on these activities on these days?** | | | | | | | | | | | | | | | | | | | | | |
|  |  | Less than 1 hour | | | |  | | 1-2  hours | | | |  | | 2-4 hours | | |  | | More than 4 hours | | |  | |
|  |  | **🗌** | | |  | | **🗌** | | | |  | | **🗌** | | | |  | **🗌** | | | | |  |

| **4. Over the past 7 days, how often did you engage in moderate sport or recreational activities such as doubles tennis, dancing, golf or other similar activities?** | | | | | | | |
| --- | --- | --- | --- | --- | --- | --- | --- |
| Never |  | Seldom (1-2 days) |  | Sometimes (3-4 days) |  | Often (5-7 days) |  |
|  | | | | | | | |
| **🗌** |  | **🗌** |  | **🗌** |  | **🗌** |  |

| **🡻** |  | |  | | **🡻** | | | | |  | | **🡻** | | | | | | |  | **🡻** | | | | | |  | |  |  |  |
| --- | --- | --- | --- | --- | --- | --- | --- | --- | --- | --- | --- | --- | --- | --- | --- | --- | --- | --- | --- | --- | --- | --- | --- | --- | --- | --- | --- | --- | --- | --- |
| Please go to next question |  | **4a On average, how many hours per day did you spend on these activities on these days?** | | | | | | | | | | | | | | | | | | | | | | | | | | | | |
|  |  | Less than 1 hour | | | | |  | | 1-2  hours | | | | | |  | | 2-4 hours | | | | |  | | More than 4 hours | | | | |  | |
|  |  | **🗌** | | | |  | | **🗌** | | | | | |  | | **🗌** | | | | | |  | **🗌** | | | | | | |  |
| **5. Over the past 7 days, how often did you engage in strenuous sport or recreational activities such as jogging, cycling, singles tennis, aerobics or other similar activities?** | | | | | | | | | | | | | | | | | | | | | | | | | | |  |  |  |  |
| Never |  | | | Seldom (1-2 days) | | | | | | |  | | Sometimes (3-4 days) | | | | |  | | | Often (5-7 days) | | | |  | |  |  |  |  |
|  | | | | | | | | | | | | | | | | | | | | | | | | | | |  |  |  |  |
| **🗌** |  | | | **🗌** | | | | | | |  | | **🗌** | | | | |  | | | **🗌** | | | |  | |  |  |  |  |

| **🡻** |  | |  | **🡻** | | | | |  | **🡻** | | | | |  | **🡻** | | | |  |  |  |  |
| --- | --- | --- | --- | --- | --- | --- | --- | --- | --- | --- | --- | --- | --- | --- | --- | --- | --- | --- | --- | --- | --- | --- | --- |
| Please go to next question |  | **5a On average, how many hours per day did you spend on these activities on these days?** | | | | | | | | | | | | | | | | | | | | | |
|  |  | Less than 1 hour | | | |  | | 1-2  hours | | | |  | | 2-4 hours | | |  | | More than 4 hours | | |  | |
|  |  | **🗌** | | |  | | **🗌** | | | |  | | **🗌** | | | |  | **🗌** | | | | |  |

**[The above questionnaire was based on PASE*. The Research Nurse reported that participants frequently told her the questions were not at an appropriate level for their degree of disability; and asked for help completing the form, which they found ambiguous. Although all responded, some said ‘Never’ to a question but then defined how many hours they spent doing it, which illustrates the problem. ]**

*****Washburn RA, Smith KW, Jette AM, *et al.* The Physical Activity Scale for the Elderly (PASE): development and evaluation. *J Clin Epidemiol* 1993;**46**:153–62.

| **ANALGESIC USE** | | | | | | | | | | | | | | | | |
| --- | --- | --- | --- | --- | --- | --- | --- | --- | --- | --- | --- | --- | --- | --- | --- | --- |
| **1. Over the last 7 days, on how many days have you taken painkilling tablets for your knee problem? (Please mark the appropriate box)** | | | | | | | | | | | | | | | |  |
| Not at all |  | 1-2 days |  | 3-4 days | | |  | 5-6 days | | |  | Every day | | |  |  |
|  | | | | | | | | | | | | | | | |  |
| **🗌** |  | **🗌** |  | | **🗌** |  | | | **🗌** |  | | | **🗌** |  | |  |

| **2. What is the average number of painkilling tablets that you take, on a typical day when you use them?** |  |
| --- | --- |

| **3. Over the last 7 days, on how many days have you used painkilling rubs or ointments for your knee problem? (Please mark the appropriate box)** | | | | | | | | | | |
| --- | --- | --- | --- | --- | --- | --- | --- | --- | --- | --- |
| Not at all |  | 1-2 days |  | 3-4 days |  | 5-6 days |  | Every day | |  |
|  | | | | | | | | | | |
| **🗌** |  | **🗌** |  | **🗌** |  | **🗌** |  | | **🗌** |  |

| **GLOBAL ASSESSMENT OF TROUBLESOMENESS** |
| --- |

| **Over the last 7 days, how troublesome has your knee pain been? (Please mark the appropriate box)** | | | | |
| --- | --- | --- | --- | --- |
| Not at all troublesome | Slightly troublesome | Moderately troublesome | Very troublesome | Extremely troublesome |
| **🗌** | **🗌** | **🗌** | **🗌** | **🗌** |

| **GLOBAL PAIN** |
| --- |

| **Over the last 7 days, thinking about any other painful areas of your body as well as your knee, how would you describe the overall pain you have experienced? (Please mark the appropriate box)** | | | | | |
| --- | --- | --- | --- | --- | --- |
| None | Very mild | Mild | Moderate | Severe | Very severe |
| **🗌** | **🗌** | **🗌** | **🗌** | **🗌** | **🗌** |

| **EXPECTATIONS OF TREATMENT FOR YOUR KNEE** |
| --- |

**On a scale where 0 is no change at all and 10 is completely better, please put a cross through the number which best describes how much you would expect your knee problem to improve with each of the following treatments:**

**1. Exercise**

Completely better

No change at all

| 0 | 1 | 2 | 3 | 4 | 5 | 6 | 7 | 8 | 9 | 10 |
| --- | --- | --- | --- | --- | --- | --- | --- | --- | --- | --- |

**2. Acupuncture**

Completely better

No change at all

| 0 | 1 | 2 | 3 | 4 | 5 | 6 | 7 | 8 | 9 | 10 |
| --- | --- | --- | --- | --- | --- | --- | --- | --- | --- | --- |

**[The Research Nurse reported that many participants expressed difficulty evaluating their expectations of change. Since 59/60 scored a response to both, there must be some doubt about the validity of these ratings].**

**END OF STUDY**

| **ANALGESIC USE** |
| --- |

| **1. Over the last 7 days, on how many days have you taken painkilling tablets for your knee problem? (Please mark the appropriate box)** | | | | | | | | | | |
| --- | --- | --- | --- | --- | --- | --- | --- | --- | --- | --- |
| Not at all |  | 1-2 days | |  | 3-4 days |  | 5-6 days |  | Every day |  |
|  | | | | | | | | | | |
| **🗌** |  | **🗌** |  | | **🗌** |  | **🗌** |  | **🗌** |  |

| **2. What is the average number of painkilling tablets that you take, on a typical day when you use them?** |  |
| --- | --- |

| **3. Over the last 7 days, on how many days have you used painkilling rubs or ointments for your knee problem? (Please mark the appropriate box)** | | | | | | | | | |
| --- | --- | --- | --- | --- | --- | --- | --- | --- | --- |
| Not at all |  | 1-2 days |  | 3-4 days |  | 5-6 days |  | Every day |  |
|  | | | | | | | | | |
| 🗌 |  | 🗌 |  | 🗌 |  | 🗌 |  | 🗌 |  |

| **USE OF EXERCISES** |
| --- |

| \| **You were given a booklet of advice and exercises in this study. How often have you done these exercises in the last 7 days?**  **(Please mark the appropriate box)** \| \| \| \| \| \| \| \| \| \| \| --- \| --- \| --- \| --- \| --- \| --- \| --- \| --- \| --- \| --- \| \| Not at all \|  \| 1-2 days \|  \| 3-4 days \|  \| 5-6 days \|  \| Every day \|  \| \|  \| \| \| \| \| \| \| \| \| \| \| 🗌 \|  \| 🗌 \|  \| 🗌 \|  \| 🗌 \|  \| 🗌 \|  \| |  |
| --- | --- | --- | --- | --- | --- | --- | --- | --- | --- | --- | --- | --- | --- | --- | --- | --- | --- | --- | --- | --- | --- | --- | --- | --- | --- | --- | --- | --- | --- | --- | --- | --- | --- | --- | --- | --- | --- | --- | --- | --- | --- |
| **GLOBAL ASSESSMENT OF TROUBLESOMENESS** | |

| **Over the last 7 days, how troublesome has your knee pain been? (Please mark the appropriate box)** | | | | |
| --- | --- | --- | --- | --- |
| Not at all troublesome | Slightly troublesome | Moderately troublesome | Very troublesome | Extremely troublesome |
| **🗌** | **🗌** | **🗌** | **🗌** | **🗌** |

| **GLOBAL PAIN** |
| --- |

| **Over the last 7 days, thinking about any other painful areas of your body as well as your knee, how would you describe the overall pain you have experienced? (Please mark the appropriate box)** | | | | | |
| --- | --- | --- | --- | --- | --- |
| None | Very mild | Mild | Moderate | Severe | Very severe |
| **🗌** | **🗌** | **🗌** | **🗌** | **🗌** | **🗌** |

| **GLOBAL ASSESSMENT OF CHANGE** |
| --- |

| **In general, how is your knee problem now compared with how it was before the start of the study? (Please mark the appropriate box)** | | | | | | |  |
| --- | --- | --- | --- | --- | --- | --- | --- |
| Much  better | Moderately better | Slightly better | No change | Slightly worse | Moderately worse | Much worse | |
| **🗌** | **🗌** | **🗌** | **🗌** | **🗌** | **🗌** | **🗌** | |
