# Supplementary figures and images for: Western medical acupuncture in a group setting for knee osteoarthritis: results of a pilot randomised controlled trial
Source: Pilot Feasibility Stud. 2016 Feb 16;2:10. doi: 10.1186/s40814-016-0051-5 (PMC5153913; doi:10.1186/s40814-016-0051-5)

## Week 1: After enrolment and randomisation

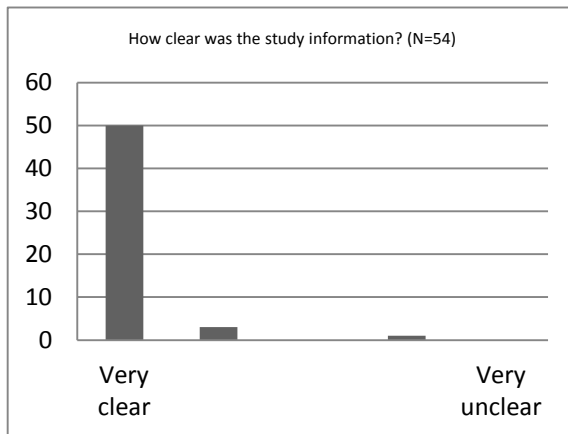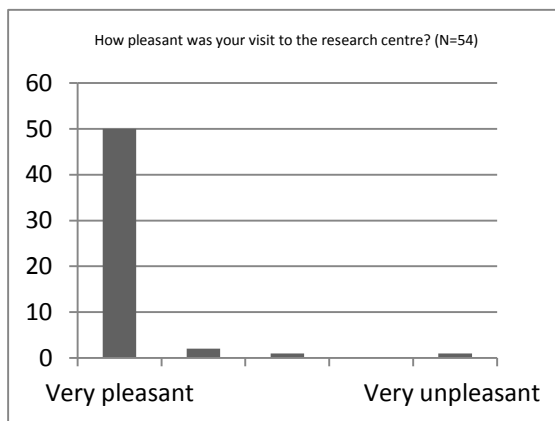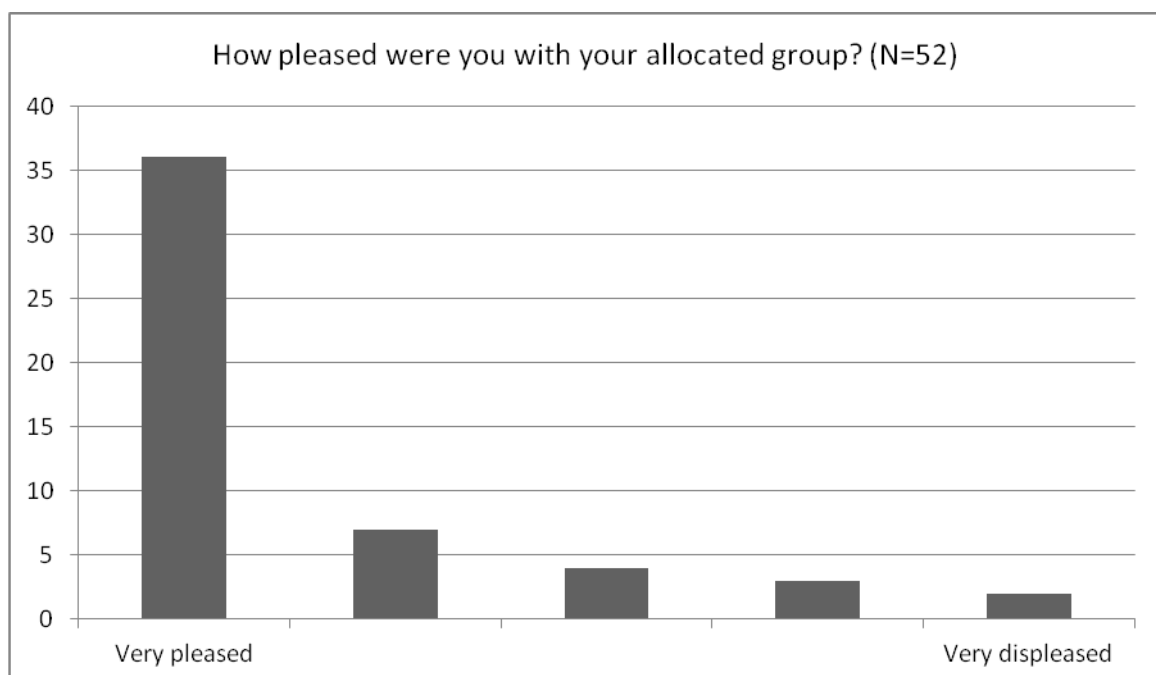

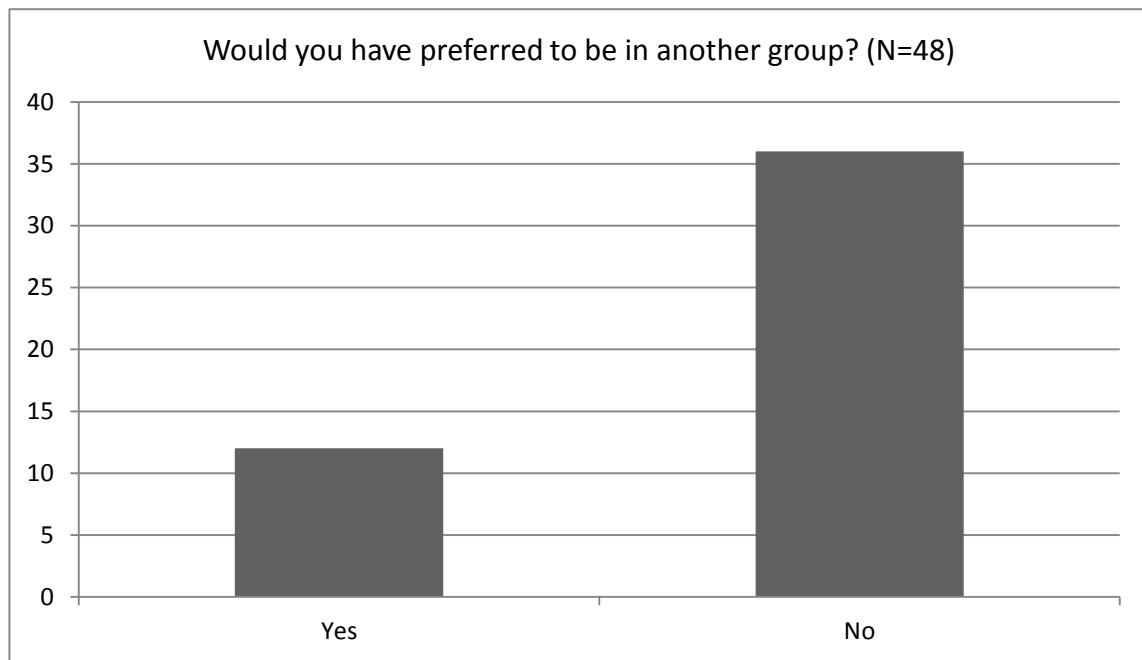

**Treatment week 2: Acupuncture groups only**

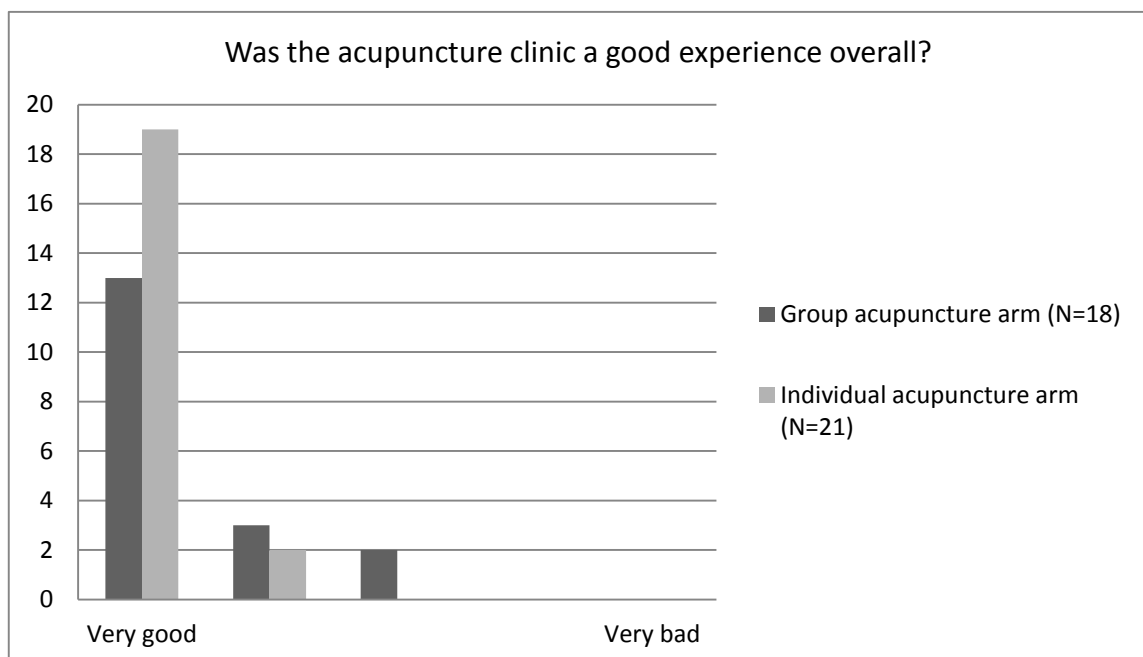

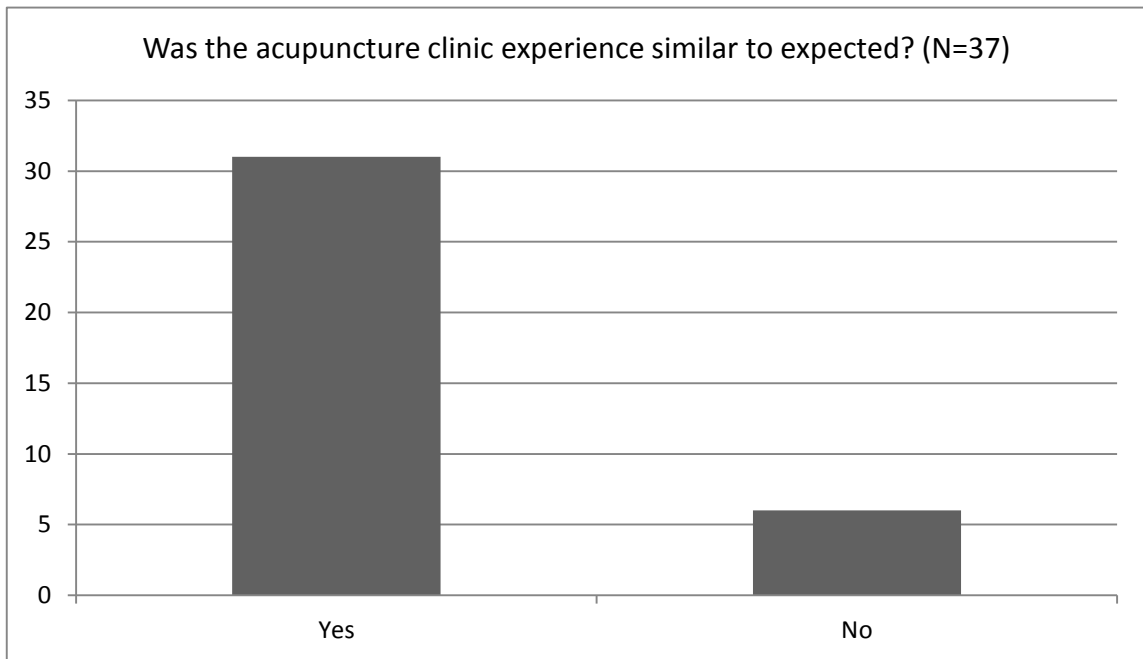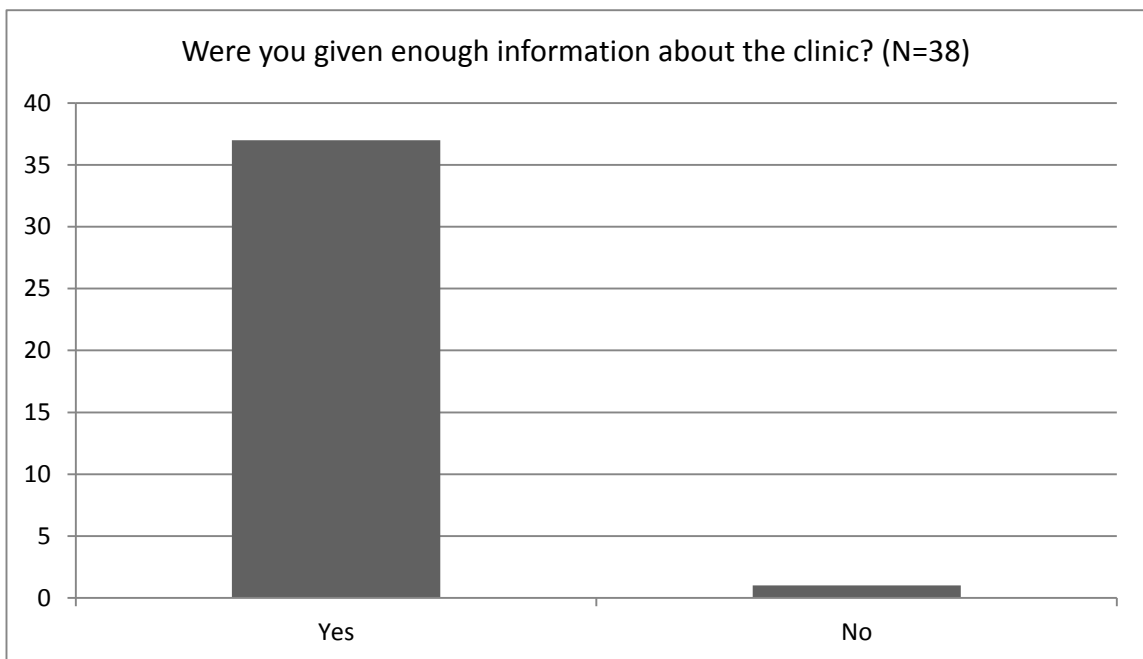

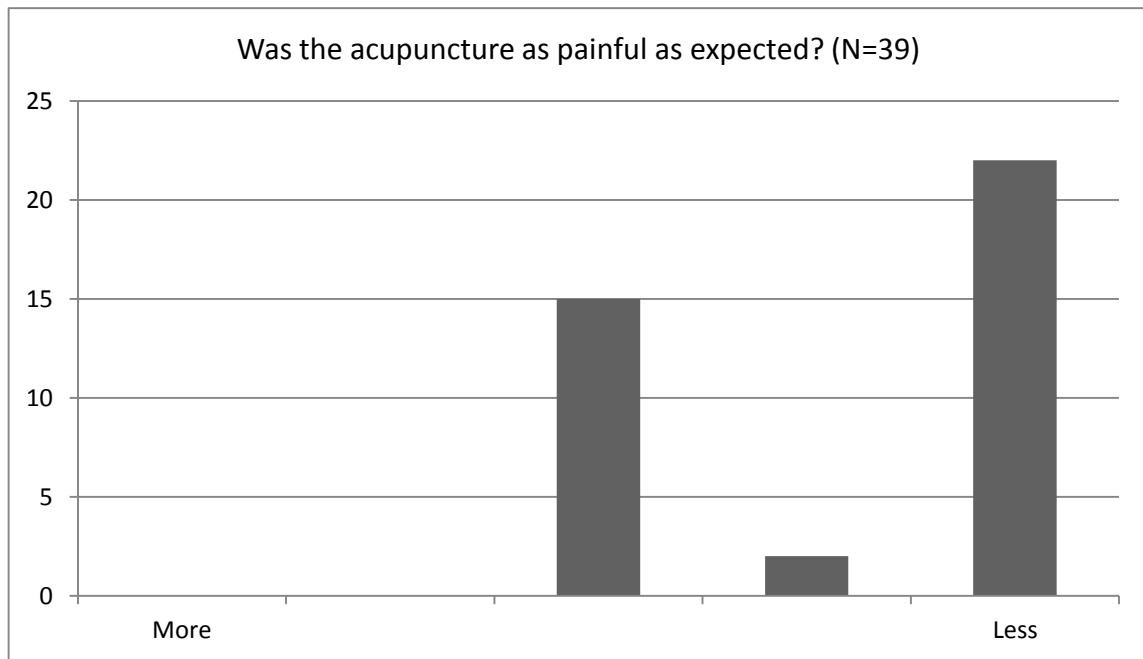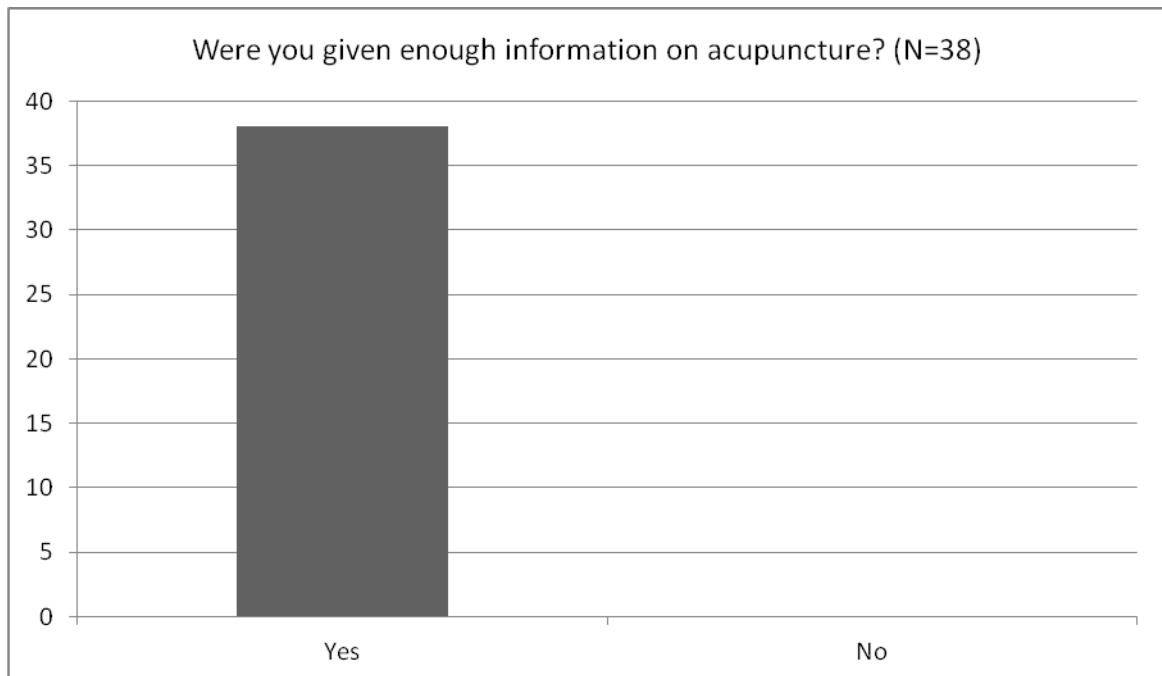

## Week 6

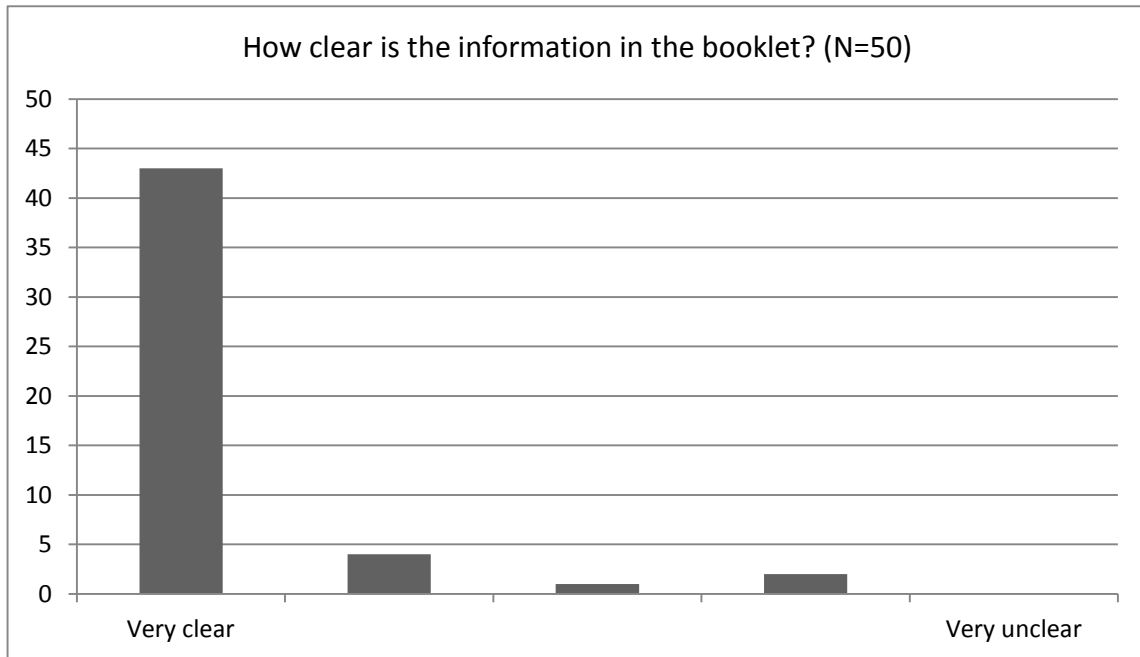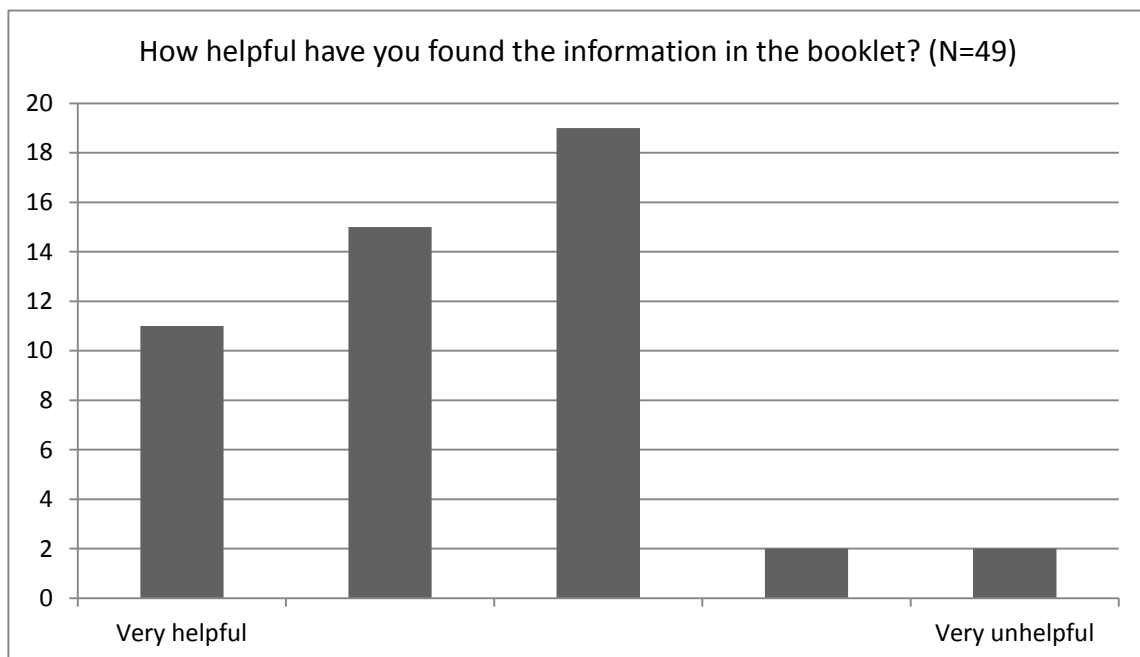

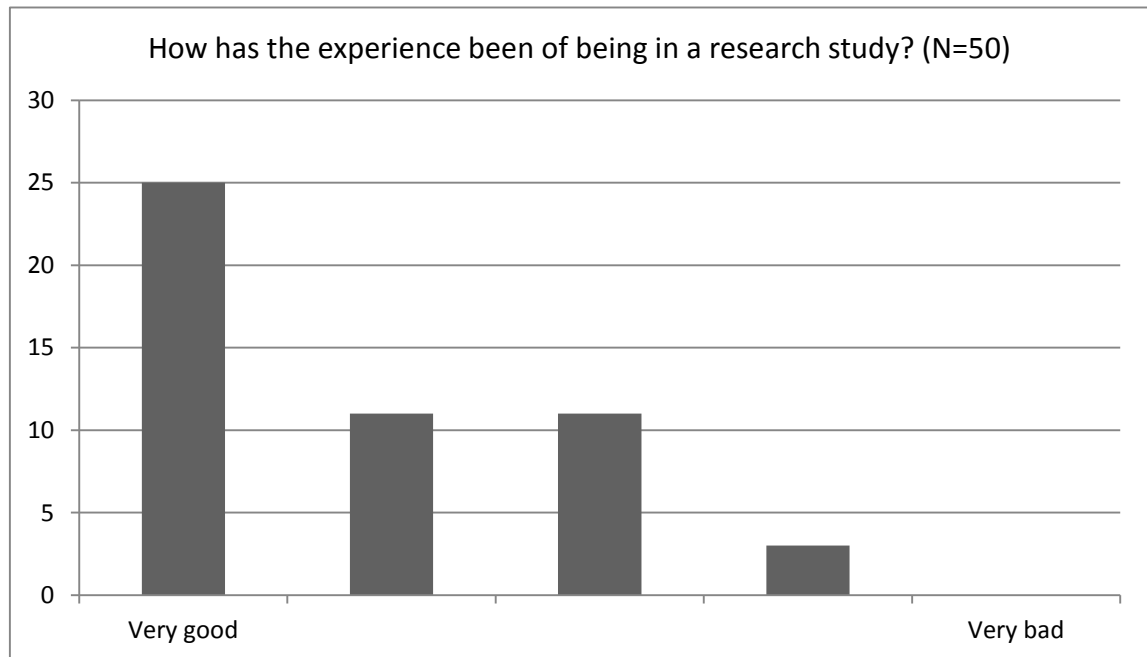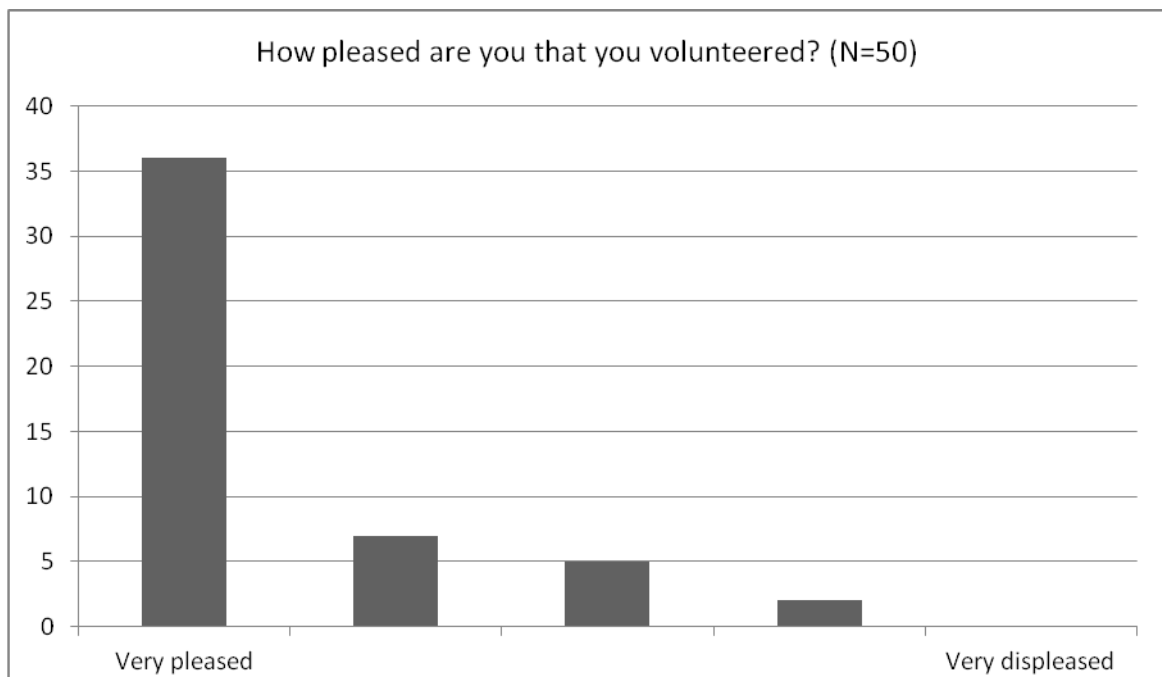

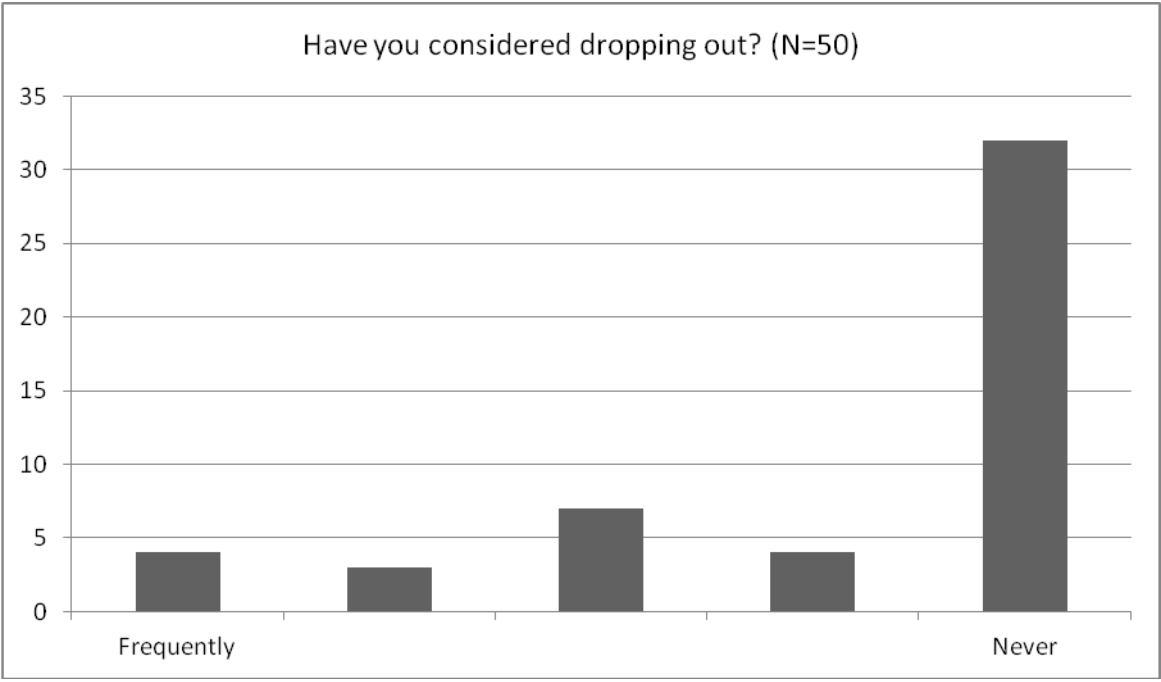

## Week 14

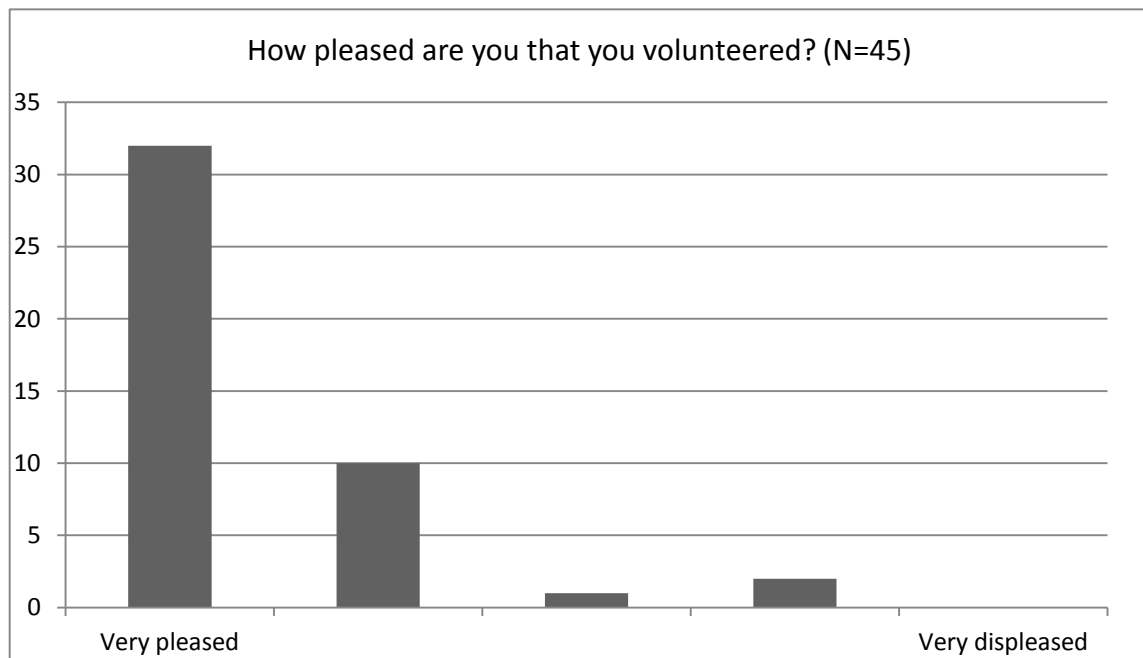

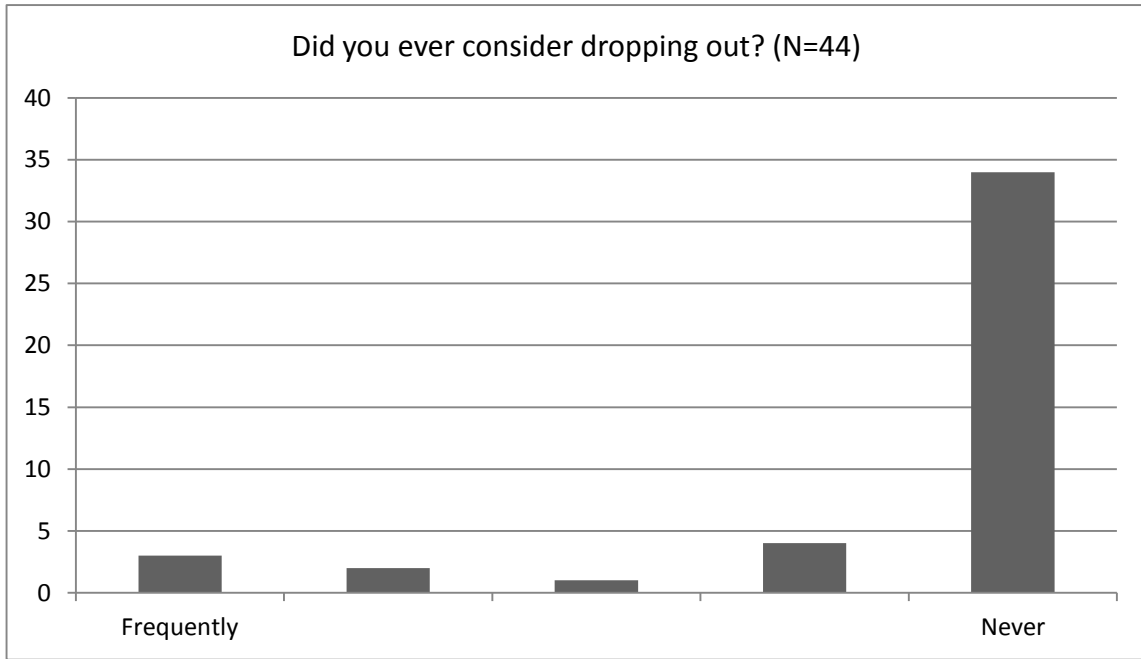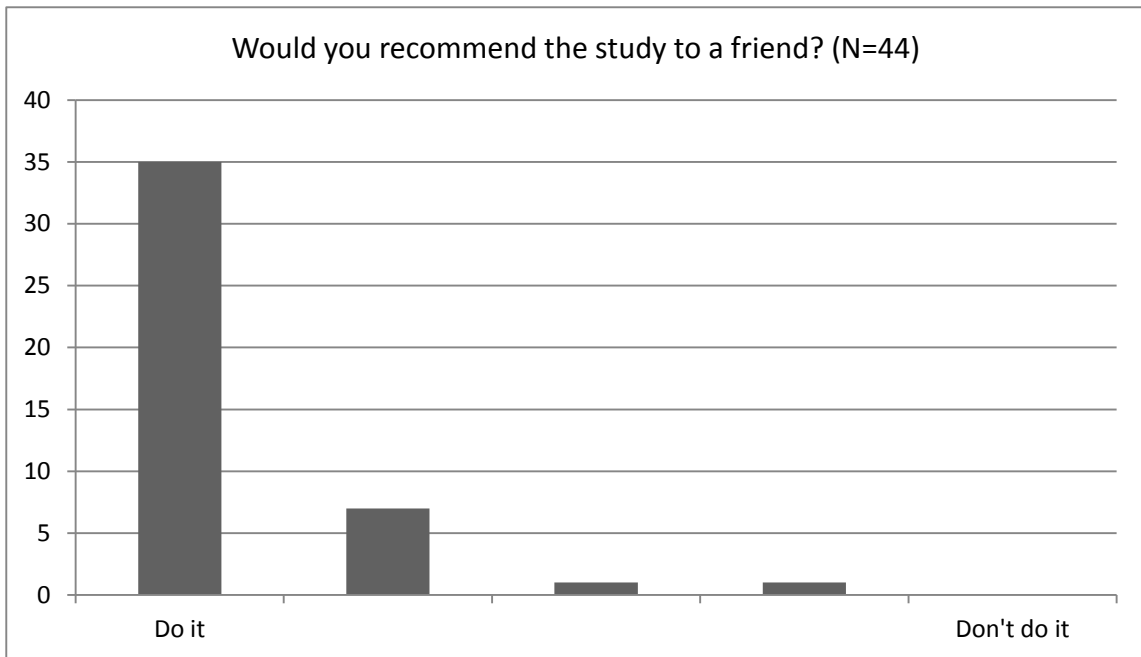

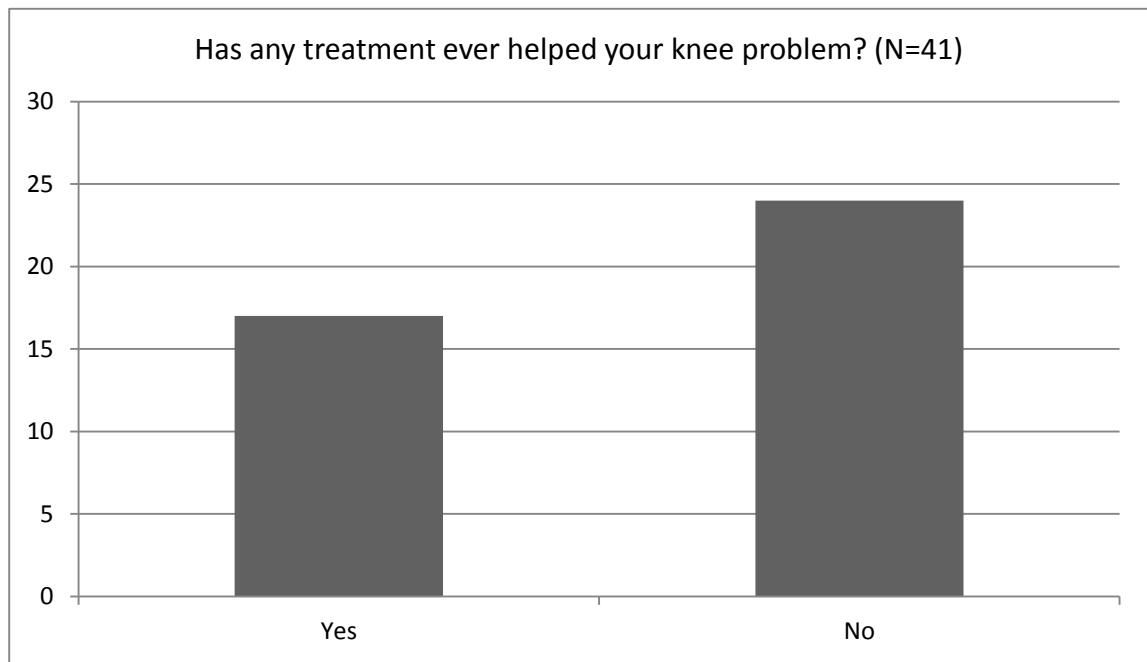

Supplement: Additional file 6: — Responses to quantitative items in ScrutiKnee commentary. (PDF 170 kb) [file 40814_2016_51_MOESM6_ESM.pdf]
